# Cancer Vaccines: Antigen Selection Strategy

**DOI:** 10.3390/vaccines9020085

**Published:** 2021-01-25

**Authors:** Yue Zhao, Alexey V. Baldin, Orkhan Isayev, Jens Werner, Andrey A. Zamyatnin, Alexandr V. Bazhin

**Affiliations:** 1 Department of General, Visceral, and Transplant Surgery, Ludwig-Maximilians-University Munich, 81377 Munich, Germany; yue.zhao@med.uni-muenchen.de (Y.Z.); alexey-baldin@belozersky.msu.ru (A.V.B.); jens.werner@med.uni-muenchen.de (J.W.); 2 Belozersky Institute of Physico-Chemical Biology, Lomonosov Moscow State University, 119992 Moscow, Russia; zamyat@belozersky.msu.ru; 3 Department of Histology, Embryology and Cytology, Azerbaijan Medical University, Baku 370022, Azerbaijan; isayev.orkhan@yahoo.com; 4 German Cancer Consortium (DKTK), Partner Site Munich, 80336 Munich, Germany; 5 Institute of Molecular Medicine, Sechenov First Moscow State Medical University, 119991 Moscow, Russia; 6 Department of Biotechnology, Sirius University of Science and Technology, 1 Olympic Ave, 354340 Sochi, Russia

**Keywords:** cancer vaccines, cancer antigens, tumor-specific antigens, tumor-associated antigens, neoantigens, cancer-germline antigens

## Abstract

Unlike traditional cancer therapies, cancer vaccines (CVs) harness a high specificity of the host’s immunity to kill tumor cells. CVs can train and bolster the patient’s immune system to recognize and eliminate malignant cells by enhancing immune cells’ identification of antigens expressed on cancer cells. Various features of antigens like immunogenicity and avidity influence the efficacy of CVs. Therefore, the choice and application of antigens play a critical role in establishing and developing CVs. Tumor-associated antigens (TAAs), a group of proteins expressed at elevated levels in tumor cells but lower levels in healthy normal cells, have been well-studied and developed in CVs. However, immunological tolerance, HLA restriction, and adverse events are major obstacles that threaten TAA-based CVs’ efficacy due to the “self-protein” characteristic of TAAs. As “abnormal proteins” that are completely absent from normal cells, tumor-specific antigens (TSAs) can trigger a robust immune response against tumor cells with high specificity and without going through central tolerance, contributing to cancer vaccine development feasibility. In this review, we focus on the unique features of TAAs and TSAs and their application in vaccines, summarizing their performance in preclinical and clinical trials.

## 1. Introduction

Since tumor antigens’ discovery, the expression pattern of which was different from normal tissues, it became obvious that tumors are immunologically distinguished from other tissues. Further, research on tumor antigens has been expanded; multiple new groups and dozens of new antigens were described. Application of tumor antigens for immunotherapy of cancer has begun. However, it has turned out that most initially described human tumor antigens are not specific for tumors; thus, they were designated as tumor-associated antigens (TAAs) and nowadays generally accepted as less suitable for being immunogens in anticancer vaccines.

Other pioneers in tumor antigens discovery are cancer-germline antigens (CGAs). Nowadays, more than 200 CGAs have been described. Later, proteins from other immune-privileged tissues were found to be expressed in tumors, such as neuronal tissue and retina. They were designated as onconeural and cancer-retina antigens, respectively. Acknowledging that such antigens can be expressed in tumors, but they are not fully tumor-specific due to their normal immune-privileged expression, they can be grouped as immune-privileged antigens. Such antigens are potential candidates for anticancer vaccine immunogens due to the absence of their expression in normal, other than immune-privileged, tissues and, therefore, high immunogenicity for the autologous immune system. Thus, by utilization of immune-privileged antigens as vaccines, central immune tolerance can be avoided. However, it is worth remembering the rare but possible autoimmune conditions due to their localization in immune-privileged tissues, similar to those in paraneoplastic syndromes.

Following antigens with incomplete tumoral specificity, tumor-specific antigens (TSAs) were described, which expression was found to be specific only for malignancies. It has been noted that tumors can be differentiated by the number of mutations that appeared for a certain period. Thus, the intensity of the mutagenic process is different in different malignancies. Such a tumor feature was designated as a tumor mutation burden (TMB). Tumors with higher TMB are more immunogenic. This phenomenon exists due to mutations and the generation of unique amino acid sequences—neoantigens—that are tumor-specific. Ironically, described as one of the first tumor antigens in mice, neoantigens are only now regaining attention as components of anticancer vaccines. A viral origin of malignancies has also been well documented. It turned out that such viral-induced malignant cells express viral antigens exclusively. Oncogenic viral antigens, thus, became of interest for anticancer immunotherapy. To summarize, there is a bright outlook regarding utilizing antigens, which will provide a strictly specific antitumor immune response, without concerns about central tolerance or, from the other side, autoimmune responses.

In the present review, we discuss prospective tumor antigens, specifically tumor-specific and immune-privileged antigens, that are worth using as anticancer vaccine components. Regarding the upcoming era of a personalized approach in medicine, a hypothetic pipeline of how to choose antigens for individual cancer treatment or preventive vaccination is also discussed.

## 2. Tumor-Specific Antigens

**Figure 1:**
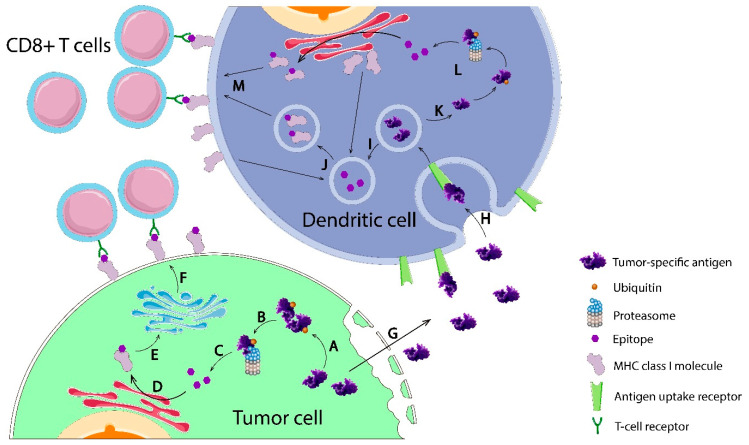
Processing and cross-presentation of tumor-specific antigens. Tumor-specific antigens (TSAs) are ubiquitinylated (**A**), transported to proteasomes (**B**), and further processed to 8–11 amino acid epitopes (**C**). After this, epitopes are loaded to MHC molecules interacting with TAP, tapasin, ERp57, and calreticulin (**D**). If the bound epitope’s affinity is high and the resulting complex is stable, the MHC–epitope complex will be transported through the Golgi apparatus (**E**) and to the cell surface for further recognition by T cells (**F**). If tumor cell disintegration happened, e.g., during necrosis, TSAs could be released into the extracellular environment (**G**), where they will be recognized and internalized via phagocytosis, pinocytosis, or receptor-mediated endocytosis by dendritic cells (**H**). Engulfed TSAs are cleaved by proteases within the endocytic compartment (primarily by cathepsins) (**I**) and further loaded into MHC molecules that are recruited either from the plasma membrane or from the endoplasmic reticulum (**J**). The alternative way of TSA processing in dendritic cells is similar to tumor cells and starts with TSA release from endosomes into the cytosol (**K**) with subsequent proteasomal degradation, including similar steps (**L**). Finally, formed MHC-epitope complexes are transported to dendritic cells’ cell surface for further cross-presentation and activation of naive T cells (**M**).

Tumor-specific antigens include antigens arisen from non-synonymous somatic mutation or viral-integrated mutation in malignant cells, completely absent from normal cells. Thus, based on (non)viral mutation etiology, TSAs can be subdivided into neoantigens and oncoviral antigens, respectively. As TSAs are expressed only in malignant cells and are foreign to the autologous immune system, the exposure of TSAs to it will induce a specific antitumor immune response. When TSAs are synthesized in cancer cells through mutations, they are exposed to the processing as other cellular proteins: being ubiquitinylated, they are transported to cytoplasmic proteasomes where they undergo proteolytic cleavage to 8–11 amino acid sequences (Figure 1). Thereafter, these peptides, called epitopes, are transported from cytosol to endoplasmic reticulum via transporter associated with antigen processing (TAP) complex, where nascent major histocompatibility complex (MHC) molecules are waiting to form an epitope-MHC complex. These complexes are then delivered via secretory vesicles to the tumor cell surface. This is a classical way of cells, including tumor cells, to “say” to cytotoxic T cells which proteins do they express. Furthermore, TSAs are released extracellularly after necrosis (Figure 1). Antigen-presenting cells (APCs) uptake and process these exogenous proteins in MHC class I and II pathways, presenting them to T cells to trigger CD8^+^ and CD4^+^ T-cell immune response. After activation, T cells mediate TSA-specific immune response, including cytotoxic response [1]. Moreover, TSAs have a higher affinity to MHC molecules and T cell receptors (TCRs) than TAAs [2]. Unlike TAAs, TSAs only presented in malignant cells but completely absent from normal cells. This feature endows TSAs a strong capability to induce a robust and specific antitumor immune response without prompting central immunological tolerance, providing a bright future for anticancer vaccine development. Currently, remarkable results have been achieved by applying TSA-based cancer vaccines in preclinical and clinical trials, with more specific, more effective, and less toxic potentials. Therefore, the search for TSAs with stronger immunogenicity has become a key and urgent issue in cancer vaccine development.

### 2.1. Neoantigens

During the process of initiation and prognosis of cancers, thousands of different genetic mutations, including single-nucleotide substitution, reading frame shift, alternative splicing, gene fusion, and other mutagenetic processes, happen in cancer cells [3]. Among these mutations, most of them do not affect tumor progression, known as passenger mutations. However, a small proportion of mutations contribute to tumorigenesis and malignization, known as driver mutations [4]. Theoretically, no matter passenger or driver mutations, they can alter the amino acid sequence, generating foreign proteins expressing in malignant but not in normal cells, collectively regarded as non-synonymous mutations [5]. Neoantigens, as a result of non-synonymous mutations, are exclusively expressed by tumor cells [2,6]. The first observation of neoantigen was made by Boon et al. in the 1980s [7,8]. They found that mutagen-treated murine P815 tumor cells produce tum^-^ mutations that are rejected by syngeneic mice because these variants express new surface antigens. These “tum^-^ antigens” were recognized by cytotoxic T lymphocytes (CTLs) but induced no detectable humoral response. Further, it has been identified that somatic mutations in cancers result in a source of neoantigens recognized by T cells in the human immune system [9,10,11].

Unlike TAAs, neoantigens exhibit a higher affinity toward MHC molecules due to their “non-self” feature and are more likely to be recognized by T cells [12]. The more considerable difference between the mutant amino acid sequence from the original sequence, the more substantial “non-self” characteristic and higher affinity to MHC molecules the neoantigens will have [13,14]. Furthermore, TCRs typically bind to neoantigens with higher affinity, and more robust T cell-mediated antitumor elimination will be induced when tumors express higher amounts of neoantigens [15,16]. The number of mutations in cancers mainly refers to TMB. Tumors with higher TMB are more likely to generate neoantigens and activate the immune system to recognize and attack tumors [17]. However, by analyzing T cells derived from tumor-infiltrating lymphocytes (TIL) or peripheral blood, data have shown that only 1.2% of screened neoantigens were spontaneously recognized by the host with melanoma, gastrointestinal, lung, and ovarian carcinomas, indicating that the frequency of neoantigens which can evoke an immune response is relatively low [18]. Thus, to achieve an effective immune-mediated tumor elimination, the search for efficient neoantigens is a principal basis in neoantigen-based cancer vaccine design.

#### 2.1.1. Sequencing and Prediction of Neoantigens

**Figure 2:**
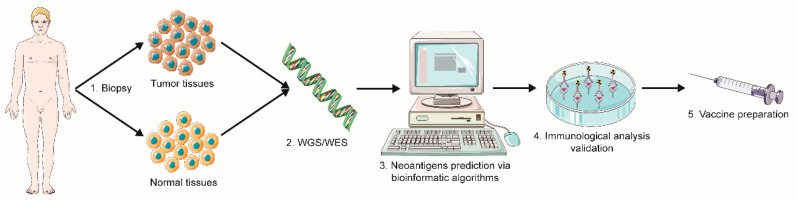
Framework for sequencing and prediction of neoantigens. 1. Biopsy is performed in cancer patients for tumor tissues and corresponding normal tissue. 2. Whole-genome sequencing (WGS)/whole-exome sequencing (WES) is carried out on tumor tissues and normal tissues to verify the somatic mutations expressed in tumor cells. 3. Bioinformatic algorithms are conducted to predict the MHC binding affinity of neoantigens, and the most attractive neoantigens for immune targeting are prioritized as candidates. 4. In vitro and in vivo experiments are used to validate the potential role of selected neoantigens. 5. Final vaccine preparation according to the selection of neoantigens. The figure was produced with the assistance of Servier Medical Art (https://smart.servier.com).

For neoantigens identification and application for cancer vaccines, several methods have been developed to distinguish neoantigens from normally expressed proteins in cancer patients. However, previous traditional cloning methods cannot meet the accuracy criteria for constructing a neoantigen-based cancer vaccine. In addition to this, inconvenient antigen processing procedure, significant consumption in time and money also contributes to the obstacle to developing neoantigens as a tumor vaccine. Thanks to the rapid progression of high-throughput sequencing technology, including whole-genome sequencing (WGS) and whole-exome sequencing (WES), neoantigen-based cancer vaccines have been more approachable for cancer patients, with higher accuracy, greater convenience, and lower cost [19]. In addition, it has been reported that WES, coupled with RNA-seq, may contribute to neoantigen prediction success. Simultaneous utilization of RNA-seq reveals information on gene expression level, allowing to predict which genes are more likely to be translated into proteins. Furthermore, RNA-seq can reveal other types of neoantigen sources, such as gene fusion, alternative splicing isoforms, and RNA editing events, that WES cannot [20,21]. Currently, for the identification and establishment of neoantigens in tumors, typically the following major steps are undertaken: (1) tumor biopsy sample and corresponding normal tissues obtainment; (2) whole-genome or -exome sequencing and analysis of mutated genes and abnormal proteins by comparison with normal tissues; (3) bioinformatic algorithms for predicting the most promising antigenic mutated protein; (4) verification of neoantigens via immunological analyses (Figure 2) [22,23,24].

Summarizing, whether mutated proteins/peptides can be recognized by the immune system and induce a robust and specific immune response mainly depends on the following factors: (1) mutated genes can be translated into proteins; (2) mutated proteins endowed antigenicity without eliciting immunological tolerance; (3) mutated proteins efficiently processed and presented by APCs; (4) high affinity of mutated peptides to MHC molecules; (5) mutated peptide-MHC complexes can be recognized by effector immune cells, mainly by T cells (high affinity to TCRs). Therefore, in the whole procedure of neoantigens searching, not only identification of neoantigens from sequencing results matters, but also affinity testing between neoantigens and MHC molecules and TCR is critical. Currently, the genomics-based approach is the most promising strategy and the gold standard in neoantigens affinity prediction. Numbers of online software, depending on the genomics-based approach and bioinformatics pipelines, are available for the prediction and identification of neoantigens [25,26,27,28,29]. For instance, the application developed by Schenck et al. named “neoantigen prediction pipeline (NeoPredPipe)” can summarize the information on predicted neoantigens burden, heterogeneity, immune stimulation potential, and, optionally, patient HLA haplotypes [29]. With these “smart” tools, neoantigens filtered from high-throughput sequencing can be used for predicting the capability of being presented on the APCs’ surface by MHC molecules and recognized by TCRs. However, this makes the genomics-based approach an imperfect strategy because it requires direct experimental evidence of predicted epitopes’ presence on the APCs’ surface in the MHC complex. Another strategy named the mass spectrometry-based approach can make up for this defect by obtaining these insufficient data via affinity chromatography [30,31]. Furthermore, structure-based approaches and neoantigen peptide databases are also unignorable supplementary methods in neoantigen prediction [20].

#### 2.1.2. Neoantigen Administration Strategies

There are three major platforms of personalized neoantigen-based cancer vaccines: synthetic peptide vaccines, dendritic cell (DC) vaccines, and DNA/RNA vaccines [32]. For vaccine manufacturing, stable storage, and reasonable cost, synthetic peptide vaccines are an ideal choice for neoantigen-based vaccines. When compared to short 8–10 amino acid peptides, which is the minimal size of peptides required to induce the activation of CD8^+^ T cells, long 15–31 amino acids peptides are more perspective in overcoming potential CTL tolerance, the involvement of Th responses, and prolongation of antigen cross-presentation [33]. A personalized neoantigen vaccine based on long synthetic peptides has already been applied in a clinical trial in melanoma patients [34].

With the rapid development and improvement of ex vivo DC culturing technologies, neoantigen-loaded DC-based vaccines have become more feasible and approachable. Vaccination of melanoma patients with DCs loaded with neoantigens has been performed [35]. In this study, exome sequencing was performed to identify somatic mutations in tumor samples, and then these mutation candidates were filtered through in silico analysis to assess the binding affinity of HLA and peptide. Seven candidate peptides were chosen and used to load DCs. After receiving the DC-based neoantigen-specific vaccine, the neoantigen-specific T-cell immune response was triggered in melanoma patients, and no severe autoimmune adverse events were observed. Same promising results were achieved in ovarian cancer patients by intranodal injection of neopeptide-loaded DC vaccine [36]. Interestingly, a recent study published by Zhang et al. demonstrated that a personalized neoantigen-pulsed DC vaccine has superior immunogenicity to neoantigen-adjuvant vaccine in murine tumor models [37].

The DNA/RNA vaccine stands out for only a small amount of tumor tissue required for amplification when preparing vaccines. Previous preclinical research utilizing a DNA vaccine to target tumor neoantigens showed that, when delivered to patients by potent electroporation-mediated DNA delivery, DNA-based neoantigen vaccines triggered a therapeutic antitumor response in vivo, and neoantigen-specific T cells expanded from immunized mice directly attacked tumor cells ex vivo [38]. However, compared with the RNA vaccine, a DNA vaccine puts patients at risk of DNA integration into the host cell genome. The concept of RNA-based neoepitope vaccine was firstly introduced by Sahin et al. [39]. After administering RNA-based neoepitope vaccines, patients with melanoma developed vaccine-induced T cell expansion and infiltration and neoepitope-specific killing of autologous tumor cells. To improve the RNA-based approach, a lipoplex-formulated RNA (RNA-LPX) vaccine, which encodes CD4^+^ T cell-recognized neoantigens, has been developed and tested in preclinical studies [40]. As compared with the radiotherapy control group, this RNA vaccine displayed a higher capability to activate more neoantigen-specific CD8^+^ T cells with lower PD-1/LAG-3 expression and a higher proportion of neoantigen-specific IFNγ^+^ CD4^+^ T cells, leading to a temporary complete remission of tumors and prolonged survival of all mice.

Recently, increasing attention has been attracted by nanovaccines due to their unique properties in cancer immunotherapy. Nanovaccines exhibit superior potentials in vaccine design and facilitation by efficient delivery to secondary lymphoid organs such as spleen and lymph nodes, high penetration of tissue barriers, tailor-designed codelivery of antigen and adjuvant, tunable intracellular vaccine release, and antigen cross-presentation in APCs [41]. Biomimetic glyconanoparticles with N-glycolylneuraminic acid, a dietary non-human immunogenic carbohydrate that accumulates on human cancer cells as a nanovaccine, have been developed and applied in mice preclinically [42]. A robust and persistent immune response was attained using this nanovaccine, resulting in inhibition of tumor growth in vivo. In the same year, a virus-like particle nano-vaccine based on a combination of CGA and mutated epitopes has been developed, revealing a promising therapeutic anticancer response [43]. Although some preclinical investigations have demonstrated the effect of nanovaccines in tumor elimination, further clinical trials are required to examine its function and efficacy, as well as biosafety.

#### 2.1.3. Clinical Progress

Application of neoantigen-based cancer vaccines in murine tumor models, including melanoma [44,45,46], glioma [47,48], pancreatic carcinoma [49], and esophageal squamous cell carcinoma [50], has shown their potential in clinical translation. With encouraging results from preclinical research, the assessment of neoantigen-based cancer vaccines in clinical trials has been boosted significantly in recent years. The first and most frequently targeted cancer type in neoantigen-based cancer vaccine clinical trials is melanoma. The initial report of a phase I neoantigen-based cancer vaccine clinical trial was released by Carreno et al. (NCT00683670) [35]. Seven neoantigens with high affinity to HLA-A*02:01 in human melanoma were sequenced and confirmed by mass spectrometry. An increased neoantigen-specific TCR repertoire in terms of both TCR-β usage and clonal composition was observed after vaccination. Later, the other two reports confirmed the great potential of a neoantigen-based cancer vaccine in struggling against melanoma (NCT01970358, NCT02035956). In the study by Ott et al., up to 20 long peptides, identified by WES data and predicted by computational algorithms, were synthesized and mixed with the Toll-like receptor 3 and melanoma differentiation-associated protein 5 agonist poly-ICLC and were used as a neoantigen-based vaccine formulation for melanoma patients [34]. Vaccination induced strong multifunctional neoantigen-specific CD4^+^ and CD8^+^ T-cell responses in melanoma patients after surgical resection. Four of six vaccinated patients had no recurrence during a median follow-up of 25 months. The other two patients with disease recurrence were subsequently treated with the anti-PD-1 antibody pembrolizumab and achieved complete responses. Unlike Ott et al., Sahin et al. developed an individualized RNA-based polyneoepitope cancer vaccine for thirteen patients with stage III and IV melanoma [39]. Ten selected mutations per patient were engineered into two synthetic RNAs, each encoding five linker-connected 27 mer peptides with the mutation. After vaccination with a maximum of 20 neoepitope vaccine doses, all patients tolerated it well without related serious adverse events. Eight patients without radiologically detectable lesions at the start of vaccination developed a robust immune response and remained recurrence-free during the 12–23 months follow-up period, while the other five patients went through melanoma relapses shortly. However, one out of five patients with recurrence achieved a complete response in combination with PD-1 blockade. Interestingly, it has been revealed in both studies that not only pre-existing weak responses against neoantigens were augmented, but also de novo responses were generated upon neoantigen-based vaccination. Moreover, both studies showed that neoantigen-based vaccines contribute to an expansion of the neoantigen-specific T cells repertoire, providing a strong rationale for the development of the neoantigen-based vaccine in clinical treatment. Although the small scale of patients in these two studies cannot fully guarantee the real efficacy of the neoantigen-based vaccine, it is not doubted that they will serve as a promising milestone and inspiring tendency for further investigation.

Glioblastoma is known as a “cold” tumor due to the immunosuppressive tumor microenvironment (TME) and low mutation rate in general. However, two recent clinical trials demonstrated a neoantigen-based cancer vaccine’s curing potential in glioblastoma treatment [51,52]. Keskin et al. manufactured vaccines that contained up to 20 long peptides admixed with poly-ICLC and administered them to immunize eight glioblastoma patients following surgical resection or conventional radiotherapy in a prime-boost schedule (NCT02287428) [51]. Patients responded well to personalized vaccines with a 7.6-month median progression-free survival and a 16.8-month overall survival, and treatment side effects were limited to grade 1–2 events. Circulating neoantigen-specific T-cells were observed in patients after vaccination. Furthermore, elevated CD4^+^ and CD8^+^ T lymphocyte infiltration were evident in patients with circulating neoantigen-specific T cells. Besides, an impressive result was found, as well as in their melanoma trial [34], that robust CD4^+^ T-cell response against immunized neoantigens that was even higher than CD8^+^ T-cell response was detected, despite the use of MHC class I binding prediction algorithms. Further optimization of algorithms may help to clarify these results as well as to enhance immunogenicity. In another work published by Hilf et al., a personalized vaccine for glioblastoma patients was designed using TAA and neoantigens as targets [52]. Fifteen patients with glioblastoma were treated with a vaccine (APVAC1), constructed from nine selected HLA-restricted non-mutated TAA peptides and one HLA class I viral marker peptide. Subsequently, 11 out of 15 patients after APVAC1 vaccination received APVAC2 composition, consists of 14 mutated and six unmutated synthetic peptides (NCT02149225). Median overall survival of 29.0 months and a median progression-free survival of 14.2 months were achieved among these patients, with mild adverse events in all patients. One patient experienced an overall survival of more than 38.9 months, with a favorable immune response pattern of CD8^+^ T and Th cell response to APVAC2 neoepitopes. These two trials revealed that neoantigen-based vaccines are feasible in glioblastoma treatment, even though low TMB and immune-suppressive TME frequently occurs in this type of cancer.

Two ongoing clinical trials of a neoantigen-based vaccine against triple-negative breast cancer (TNBC) were reported by Li et al. (NCT02427581, NCT02348320) [32]. Patients with TNBC who did not achieve a complete response after neoadjuvant chemotherapy were included in these two trials. In one of them (NCT02427581), neoantigen vaccines were designed in synthetic long peptide form admixed with poly-ICLC. In the second trial (NCT02348320), the neoantigen polyepitope DNA vaccine was manufactured and administered to patients by electroporation. During these trials, the efficacy and safety of the personalized neoantigen vaccine will be assessed. Neoantigen-specific T-cell response will be detected during pre- and post-vaccination periods. Furthermore, an increasing number of clinical trials to assess neoantigen-based vaccines in various types of cancers are underway (Table 1). Summarizing, although the number of clinical trials concerning neoantigen-based vaccines is limited, neoantigen cancer vaccines showed promising effectiveness in triggering neoantigen-specific T-cell response among cancer patients, as well as promoting clinical remission.

#### 2.1.4. Combination with Other Therapies

Although personalized neoantigen-specific vaccines trigger a T-cell response against malignant cells, immune evasion is inevitable due to multiple immune escape mechanisms possessed by tumor cells. Previous research discovered that in murine models, after receiving neoantigen-based vaccination, a vast majority of specific TILs co-expressed PD-1 and TIM-3 molecules, which are inhibitory receptors associated with immune suppression [53]. High levels of inhibitory receptor expression on T cells induced by neoantigen-based vaccines accelerate T cells’ exhaustion and dysfunction, resulting in a weaker immune response against tumors. It is known from available clinical trial data that the objective response rates (ORRs) to immune checkpoint blockade (ICB) correlate positively with somatic mutation frequency in tumors [2]. Patients bearing tumors with high TMB, like melanoma and non-small-cell lung carcinoma (NSCLC), had higher ORRs to PD-1 and PD-L1 inhibition, whereas patients bearing tumors with lower TMB, such as Ewing sarcoma and prostate carcinoma, demonstrated fewer responses to ICB treatment. These data strongly support the approach of neoantigen-based vaccination combination with ICB therapy. Furthermore, a recent study reported that neoantigens could induce T-cell immunoreactivity and sensitivity to ICB, underlying the reason tumors with high TMB respond well to ICB [54]. In the report by Ott et al. mentioned above, where two out of six melanoma patients were still suffered poor therapeutic effects after vaccination with synthetic neoantigen peptides, their complete anticancer responses were observed after treatment with the PD-1 blockade [34]. Similar effects were also evident by Sahin et al. [39]. Their results highlight the value of neoantigen-based vaccines in combination with ICB in cancer clinical trials. Additionally, a phase Ib trial report released recently by Ott et al. strengthens the prospects of combining neoantigen-based vaccines and ICB in cancer treatment (NCT02897765). They applied a neoantigen-based vaccine consists of up to 20 unique peptides and mixed it with the adjuvant poly-ICLC for administration into patients with unresectable or metastatic melanoma, smoking-associated NSCLC, and urothelial carcinoma of the bladder. Patients were treated with nivolumab for 12 weeks in advance while the vaccines were generated. After five priming and two booster vaccinations with continuous nivolumab administration, of the 60 patients, the ORRs were 59%, 39%, and 27%, whereas the RCRs were 15%, 17%, and 7% for melanoma, NSCLC, and bladder carcinoma patients, respectively. The median PFS among vaccinated patients was 23.5 months, 8.5 months, and 5.8 months, respectively. These data showed that a personalized neoantigen-based vaccine, when co-administered with anti-PD-1 therapy, is practicable in various solid tumor types. Moreover, neoantigen-based vaccine, in combination with anti-PD-1, generated cytotoxic T cells that can reach metastatic tumors and induce epitopes spreading, expanding the repertoire of T-cell response against tumor neoantigens. Summarizing the results above, a strong rationale that neoantigen-based vaccine, in combination with ICB, could augment the response rates and durability of responses over the monotherapy was achieved.

The strategy of neoantigen-based vaccines combined with traditional cancer therapies, such as chemotherapy and radiotherapy, still cannot be neglected for its enhancing therapeutic effect in cancer patients. When applying chemotherapy or radiotherapy for cancer treatment, enhanced antigens release from tumor cells was evident, enabling their recognition by APCs and T cells for a more active immune response [55]. Certain tumors with low TMB express a low number of neoantigens and possess low neoantigen-specific immunoreactivity, therefore being an unsuitable target for neoantigen-based vaccines. Nonetheless, it is possible to overcome this problem by combining neoantigen-based vaccines with chemotherapy or radiotherapy. Increasing evidence showed that conventional therapy augments the antigenicity and immunogenicity by altering the neoantigen repertoire, increasing neoantigens generation and expression, and activating T cell trafficking and reactivity [56,57]. Some ongoing phase I clinical trials concerning neoantigen-based vaccines combined with chemotherapy is established to explore the efficacy and safety of cancer treatment. For instance, a pilot study reported by Bachireddy et al. aimed at exploring the effectiveness of the neoantigen-based vaccine in combination with low-dose cyclophosphamide being administered in a priming and booster phase in lymphocytic leukemia patients (NCT03219450). However, more studies are urgently needed to discuss the potency of neoantigen-based vaccines combined with conventional therapy strategies in cancer treatment.

### 2.2. Viral Antigens

It has been reported that almost 15% of human cancers are caused by viral infection [58]. Experimental evidence and epidemiological data revealed that human viruses critically participate in malignant disease occurrence and development. There are three major kinds of tumor-associated viruses: retroviruses, non-retroviral RNA viruses, and DNA viruses. The mechanisms of viral-mediated carcinogenesis vary significantly among different types of viruses. For instance, some retroviruses transduce viral oncogenes in host cells or integrate viral genes in the vicinity of cellular proto-oncogenes, resulting in cellular transformation and stimulation of cell proliferation directly [59]. On the other hand, chronic inflammation, alteration of the immune response, accumulation of mutations, or chromosomal alteration in infected cells constitutes the indirect mechanisms of viral-mediated carcinogenesis. During the initiation and progression of viral-associated cancers, some viral oncoproteins are expressed continuously in virus-infected cancers or precancerous lesions, but not in normal healthy tissues [2]. The recent rapid development of next-generation sequencing technologies and integrated expression profiling analyses contributes to identifying viral antigens in viral-related cancers. Being TSAs that absent in normal cells, viral antigens are endowed with a powerful capability to induce cellular immune response mediated by CD4^+^ and CD8^+^ T lymphocytes to eliminate virus-associated tumor cells [60]. Moreover, humoral immunity against targeted viral antigens will also be trigger after stimulation, leading to antibody production and neutralization of antigens, which is the basic concept of prophylactic vaccine development. Thus, further exploration of viral antigens will provide prospective candidates for cancer antigens selection in cancer vaccine development. Hereby, several suitable viral antigens and their specific target cancer types are discussed below.

#### 2.2.1. Human Papillomavirus and Cervical Carcinoma

Human papillomaviruses (HPVs) are small DNA viruses that in humans mainly infect epithelial tissues and mucosa [61]. Currently, more than 200 different HPVs have been discovered [62]. Among these HPVs, the high-risk types, such as HPV 16, 18, 31, 33, etc., are identified, and infection with them is known to correlate with a high chance of squamous lesions and progression of cervical and other anogenital carcinomas [63]. Noteworthy, up to 70% of oropharyngeal carcinomas harbor the DNA of HPVs [64]. The genome of HPVs is presented by circular double-stranded DNA, which is approximately eight kbp. It can be divided into long control region (LCR), early region (E), and late region (L) by their biological functions [65]. The LCR comprises almost 10% of HPVs genome; E encodes six to eight poly-function proteins; L encodes two structural proteins. Among all these genes, E6 and E7 are distinguished for their constant expression in most cervical cancers and cellular pro-transformation function. It has been well studied that E6 acts as an oncoprotein by promoting the degradation of the tumor suppressor p53, while E7 fulfills its oncogenic ability via binding to the retinoblastoma susceptibility protein (pRb) and causing its degradation [66,67]. Being TSAs, E6 and E7 can trigger a robust tumor-specific immune response [68,69]. Furthermore, the infiltration of CD8^+^ T cells specific to E6 and E7 proteins was evident in the spontaneous regression of cervical intraepithelial neoplasia (CIN) [70,71]. Thus, E6 and E7 have been recognized as prospective target proteins for developing HPV-related cancer vaccines, especially for cervical cancer. Based on this strategy concept, several E6- or E7-based cancer vaccines were investigated according to different platforms, including bacterial vector, viral vector, DNA-, peptide-, and DC-based vaccines [72,73,74,75,76]. It has been shown that HPV 16 E5 induces cellular transformation in primary cells by promoting the effects of E6 and E7 [77]. E5 also showed potential in regulating epidermal growth factor receptor and platelet-derived growth factor receptor [78]. Roles of HPV 16 E5 in cervical carcinogenesis were summarized and revealed that HPV E5 oncoprotein could serve as a new target for cervical cancer treatment [79]. Furthermore, the application of HPV 16 E5 peptide in cancer vaccine formulation was efficient against cervical cancer in vitro and in vivo. Results showed that robust cellular immunity was activated after vaccination, and mice were protected from tumor growth [80]. Summarizing, oncoproteins E5, E6, E7 derived from HPVs are the most applied targets for HPV-related tumors and have been frequently employed in cancer vaccines with promising outcomes.

#### 2.2.2. Hepatitis Viruses and Hepatocellular Carcinoma

Hepatocellular carcinoma (HCC) is one of the most frequent and malignant diseases worldwide. Infection with hepatitis virus type B (HBV) or hepatitis virus type C (HCV) has been proven to correlate directly with HCC onset. HBV is a double-strand DNA virus with a genome of approximately 3.2 kbp, which harbor four overlapping reading frames [81]. There are three primary mechanisms by which HBV induces carcinogenesis: (1) HBV proteins are involved in cellular signing pathway; (2) integration of HBV DNA into the host genome; (3) virus-medicated chronic inflammation [82]. In the first two mechanisms, HBV-related oncoproteins are generated and participated in the tumorigenesis, which could be used as targets for immune cells’ recognition. The most well-identified oncoprotein HBV X (HBx), encoded by the *X* gene, has been demonstrated to be involved in signaling pathways, like MAPK and JNK, to promote proliferation and interfere with DNA repair mechanisms, contributing to a malignant phenotype eventually [83,84,85]. Based on HBx oncoprotein, several vaccines against HBV-related HCC were explored for the therapeutic efficacy against HCC in vivo and in vitro. Ding et al. inserted multiple HBx epitopes into HBV core protein to form multiepitope peptide-loaded virus-like particles (VLPs). HBx-specific CTL immune response was triggered, and a positive antitumor effect in mice was observed after vaccination with VLP-pulsed DCs [86]. A novel recombinant *Listeria monocytogenes* (LM)-based vaccine containing HBx epitope fragments for HCC treatment was reported by Chen et al. [87]. T-cell proliferation and enhanced supernatant level of interferon-γ in vitro were detected following vaccination. Moreover, interferon-γ-producing CD8^+^ T cells as well as in vivo cytolytic activity were significantly increased. These results revealed that HBx could be a prospective target antigen in establishing an HBV-related cancer vaccine.

HCV harbors a single strand RNA of approximately 9.6 kbp and encodes a poly-protein precursor, from which smaller structural and nonstructural proteins are generated by cleavage [58]. Three molecules, namely HCV core, NS3, and NS5A, even though they have not been proved to be direct oncogenes during HCV-related carcinogenesis, exhibit transformation potential and take part in cell signing pathway, including transcription, proliferation, and apoptosis, indicating their potential cancer vaccine components [88,89,90]. It has been reported that a multi-peptide cocktail vaccine containing both HCV NS3 and core epitopes, in combination with metronomic chemotherapy, was applied to struggle against HCV-related HCC [91]. An overall increase in IFN-γ and IL-4 producing CD8^+^ T cells in mice was evident when treated with daily metronomic chemotherapy and multi-peptide vaccine. The further multiparametric analysis confirmed an enhanced antigen-specific immune response and Treg depletion triggered by HCV epitope-based vaccine if administered with daily metronomic chemotherapy. Nevertheless, more studies about HCV-related epitope-based vaccines in cancer treatment are required to prove their efficacy.

#### 2.2.3. Epstein–Barr Virus and Nasopharyngeal Carcinoma

Epstein–Barr virus (EBV) is an oncogenic γ herpesvirus which infection rate over 90% of the adult population worldwide [92]. EBV is commonly transmitted via contact with respiratory secretions and then enters into the upper respiratory tract’s reticuloendothelial cells. However, B lymphocytes are the primary target cells of EBV after virus dissemination throughout the host body. EBV has been associated with nasopharyngeal carcinoma (NPC), Burkitt lymphoma (BL), and Hodgkin lymphoma (HL). Notably, almost all NPC cases are EBV-associated, and the infections are predominantly in the latent phase [93]. EBV, with an approximately 180 kbp genome, harbors more than 90 genes. The most well-identified genes are latent membrane proteins (LMPs) and EBV nuclear antigen 1 (EBNA1), for their pivotal roles in carcinogenesis. Thus, targeting of these two oncoproteins by vaccines is aimed to struggle against EBV-related tumors. In 2003, a preclinical study described a recombinant poxvirus vaccine that encodes LMP1 epitopes [94]. This polyepitope vaccine consistently generated a strong LMP1-specific CTL response and successfully reversed the outgrowth of LMP1-expressing tumors in mice. A recombinant adeno-associated virus, carrying a fusion gene containing EBV LMP1 and LMP2 CTL epitopes and heat shock protein as an adjuvant, has been investigated as a potential vaccine for NPC treatment [95]. After vaccination, mice exhibited a tumor elimination trend and achieved extended survival time. LMP1/2-specific CTL responses, as well as humoral immune response, were induced to decelerate tumor growth. Another recombinant vaccine named MVA-EL, which is modified vaccinia Ankara virus, encoding CD4 epitopes of EBNA1 and full-length LMP2 was developed [96]. After stimulation of DCs with this vaccine, EBNA1 and LMP2 were processed via HLA class I and class II pathways and presented to T cells, resulting in an expansion of T lymphocytes, especially T memory cells. A similar strategy was also performed in another study, where the vaccine represented a fusion protein incorporating random overlapping peptides from EBNA1, LMP1, and LMP2, which was then inserted into a replication-deficient strain of adenovirus [97]. After stimulation with this vaccine, activation and expansion of EBNA1- and LMP-specific CTL responses were observed in healthy donors and NPC patients, revealing the potential role of EBNA1- and LMP-based vaccine in NPC treatment.

#### 2.2.4. Clinical Advance of Viral Antigen-Based Cancer Vaccines

Prophylactic viral vaccines, such as HPV vaccine and HBV vaccine, are clinically available at an approachable cost, preventing individuals from getting viral infections and further virus-related carcinogenesis. However, prophylactic HPV vaccines aim to induce immunity against structural viral antigens. For instance, current prophylactic HPV vaccines mainly aim to induce immunity against L1 capsid protein and provide limited therapeutic benefit due to L1 protein eventually being expressed only in granular epithelium just prior to viral shedding [98,99]. By contrast, the current HPV virus-associated cancer vaccine, as a therapeutic vaccine applied in clinical trials, eradicate cancer or precancerous lesions mainly via E6- and E7-specific immune response. Since in the present review we are focusing on antigens that are (aberrantly) expressed in cancer cells, prophylactic vaccines against viruses will not be further emphasized. Although vaccines for preventing viral infections are considered prophylactic for cancer diseases in viral-induced cancers, vaccines containing viral antigens that start to express in malignant cells can also be applied as prophylactic agents for preventing relapse after tumor resection. Moreover, in this case, it is not necessary to focus only on viral antigens. However, despite different purposes, prophylactic or therapeutic, the composition of such vaccines will be the same since they are aimed to induce immunity against malignant cells expressing known antigens of the known tumor, resected or not. Previous preclinical studies already revealed the potential capability of viral antigen-based cancer vaccines in cancer treatment. Thus, to have a clear understanding of viral antigen-based cancer vaccines’ performance in clinical trials, several existing data are summarized in Table S1.

Viral antigen-based cancer vaccines against cervical cancer mainly target HPV 16 E6/E7 molecules. Two trials reported by Welters et al. and Maciag et al. showed that HPV 16 E6/E7 targeting vaccines were applied in six and fifteen patients with cervical carcinoma and achieved 13 and 11.6 months OS periods, respectively [100,101]. Welter et al. developed a peptide vaccine that triggers a specific T-cell immune response in all patients, with no serious adverse events. In contrast, the specific T-cell immunity was observed in 1 out of 3 patients in Maciag’s trial by applying Listeria monocytogenes vaccine for cancer treatment, with six grade 3 adverse events occurred among patients. However, seven patients achieved stable disease status while one patient fulfilled a partial response after vaccination in Maciag’s trial. Furthermore, apart from cervical cancer, HPV antigen-based vaccines were also employed in other cancer types. Aggarwal et al. utilized a DNA vaccine encoding HPV 16/18 E6/E7 to struggle against head and neck squamous cell cancer (HNSCC) [102]. This DNA vaccine induced HPV antigen-specific T-cell response in eight out of 21 patients and benefitted HNSCC patients with a 15.9 month OS time, with only mild adverse events.

Besides, EBV antigens are frequently utilized in cancer vaccines developed for NPC specifically, which is usually associated with EBV infections and begin to express EBV antigens during carcinogenesis. In 2002, the first EBV antigen-based vaccine targeting LMP2 for NPC treatment was investigated by Lin et al. [103]. By stimulating DCs with LMP2 peptides, this DC vaccine was administrated into sixteen NPC patients and bolster a peptides-specific immune response in nine patients. Two patients experienced a partial response and showed a typically strong immunological response to vaccination. A subsequent phase II clinical trial reported by Chia et al. further confirmed the therapeutic capability of EBV antigen targeting vaccine for NPC therapy [104]. Among eight patients who received a vaccination, two showed stable disease status, and one achieved partial clinical response, whereas only one grade 3 case of adverse event was reported, demonstrating its therapeutic potential and safety. Regrettably, no specific T-lymphocytic response was detected in these patients. Furthermore, a therapeutic NPC vaccine comprising a recombinant vaccinia virus, MVA-EL, was designed to boost the immunity targeting EBV EBNA1, LMP2 by two cooperating groups from Hong Kong and the United Kingdom [105,106]. Combined results suggested that specific EBNA1- or LMP2-T-cells immune activities were detected in 23 out of 32 patients, and only one patient went through a grade 3 adverse event. Interestingly, a dose-dependent immune response was observed by ELISpot assays in the absence of dose-limiting toxicity. Taken together, EBV antigen-based vaccines were showed to be safe and, to a certain extent, effective in NPC clinical treatment.

Although preclinical research has shown the possibility of applying hepatitis virus antigens in the development of HCC vaccines, the employment of hepatitis virus antigen-associated vaccine was rarely reported in clinical trials, mainly due to the high mutation rates in HBV and HCV. One available phase II clinical trial reported by Yutani et al. showed a peptide vaccine consisting of a single HCV-derived peptide of a core protein and 31 peptides derived from 15 different TAAs, targeting HCV-positive advanced HCC [107]. This vaccine elicited a specific T-cell immune response in 19 out of 36 patients while induced fourteen cases of grade 3 adverse events and one case of a grade 5 adverse event among all patients. After vaccination, the median OS time of a total of forty-two patients was 6.1 months. Noteworthy, when combined with sorafenib therapy, this therapeutic cancer vaccine had no difference in OS compared with the vaccine alone. Therefore, the selection of viral antigens without high mutations for utilization in cancer vaccine formulations and making them more feasible for large-scale clinical trials are still future challenges for viral antigen-based cancer vaccine development. Another challenge for vaccine development is the choice of platform for antigen or antigen-encoding genetic material delivery. As discussed earlier in the neoantigen section, mRNA vaccines look promising with their positive attributes (induction of both humoral and cytotoxic immunity, rapid manufacturing, safety, absence of vector-specific immunity) and recent advances in RNA-based COVID-19 vaccines [108,109]. Thus, mRNA-based vaccines’ success may be translated for the development of virus-associated antigen-encoding mRNA vaccines to struggle with malignant diseases.

## 3. Tumor-Associated Antigens

Tumor-associated antigens (TAAs) are antigens that are overexpressed in malignant cells but also presented in normal cells at a low level of expression. The aberrant expression of TAAs in tumors usually results from genetic amplification or post-translational modifications [2]. There are three major types of TAAs: overexpressed antigens, differentiation antigens, and CGAs. Overexpressed antigens are “self-proteins” moderately expressed in most healthy tissues but abundantly in tumor tissues. As a typical and well-established representative of overexpressed antigens, HER-2/neu is found to be expressed in most normal epithelial cells and various tumor types, especially in breast carcinoma [110]. Other TAAs are selectively expressed by the cell lineage from which malignant cells evolved. These antigens were designated as differentiation antigens. One representative example, prostate-specific antigen (PSA), has a highly restricted tissue distribution and is expressed in normal epithelial cells of the prostate gland, as well as prostate carcinomas [111]. The application of overexpressed and differentiation antigens in cancer vaccine development has been well discussed and summarized before [112,113,114]. These two kinds of tumor antigens are currently proved not to fit well in the landscape of cancer vaccines, with high immunological tolerance and toxicity that threaten the efficacy and safety of cancer vaccines for patients. Furthermore, current trends in cancer vaccine development are focusing more on personalized and accurate approaches. Therefore, overexpressed antigens and differentiation antigens will not be emphasized in our review. As for the CGAs, they stand aside among TAAs. Besides their nonspecific tumor expression, they have only been found to be expressed in immune-privileged tissues. Thus, their aberrant expression in tumors makes them highly immunogenic. In addition to segregation by tissue barriers, trophoblastic and male germ cells, which are normal localization of CGAs, lack HLA class I molecules expression and therefore cannot present antigens to T cells [115,116,117]. Although considered TAAs, such features of germline cells make CGAs insignificant to potential autoimmune response but a potential target in cancer immunotherapy.

### 3.1. Cancer-Germline Antigens

**Figure 3:**
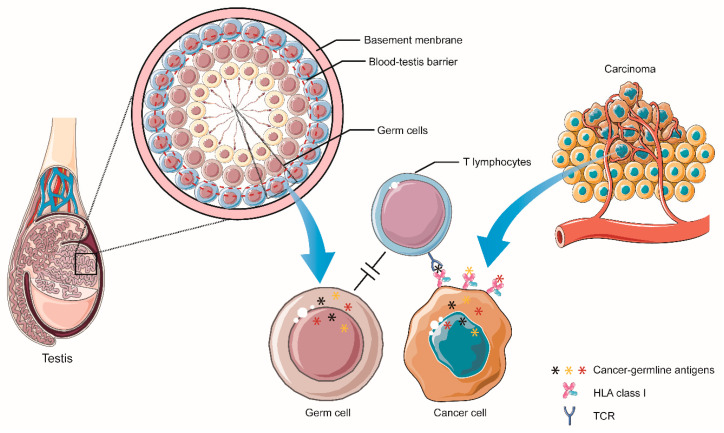
The immunological nature of cancer-germline antigens. The expression of cancer-germline antigens (CGAs) is normally limited to reproductive tissues. The presence of the blood-testis barrier makes testis an immune-privileged zone, which means CGAs expressed in germ cells do not interact with the immune system. Furthermore, due to the lack of HLA class I molecules on the surface of germ cells, CGAs cannot be presented on their cell surface, making germ cells unrecognizable for CGA-specific immune cells. These features make CGAs foreign when exposed to the immune system and endow them with a strong capability to induce a robust immune response. Aberrant expression of CGAs can be detected in various cancers. These antigens, being expressed in malignant cells, will be processed and presented on the surface of malignant cells, including the HLA class I pathway. Once recognized by immune cells, CGAs will be targeted by the immune system, leading to the destruction of CGA-expressing malignant cells.

CGAs expression is restricted to reproductive tissues. Due to the presence of blood-testis barrier and devoid expression of HLA class I molecules on the surface of germ cells, CGAs are capable of avoiding interaction and recognition by the immune system when expressed in germinative tissues, which are known as immune-privileged zones (Figure 3) [118]. However, accumulated data have revealed that CGAs are expressed in various kinds of cancer ectopically, mainly due to epigenetic regulation activities, including DNA methylation and histone post-translational modification [119]. Furthermore, CGAs play a critical role in the initiation and progression of cancers. Immune-privileged nature endows CGAs a strong ability to elicit an immune response when expressed in tumors due to decreased, even absent, peripheral tolerance. Cytotoxic and humoral immune responses to CGAs are frequently evident in cancer patients, and CGAs’ aberrant expression in tumors has been linked with disease stage, worse clinical outcomes, and cancer invasion [120,121]. Together, restricted expression in germinative tissues, ample aberrant expression in various cancer types, and immunogenic nature make CGAs promising targets for cancer vaccine development. The first identified CGA was melanoma-associated antigen 1 (MAGE-1), discovered by Bruggen et al. via autologous typing with T cell clones from a melanoma patient with advanced cancer stage [122]. According to available information, currently, there are more than 200 CGA genes in the human genome, classified into 44 gene families [123]. Among all CGA candidates, melanoma-associated antigen A3 (MAGE-A3), New York esophageal squamous cell carcinoma-1 (NY-ESO-1) antigen, and preferentially expressed antigen in melanoma (PRAME) have been well-studied and proved to be promising cancer vaccine targets. Therefore, these three CGAs and other current promising targets are included for discussion in our review.

#### 3.1.1. Melanoma Antigen Family A3

MAGE-A3 belongs to a gene family containing more than 60 members, almost all located on the X chromosome [124]. A conserved MAGE homology domain is shared in all MAGE-A family proteins, while no noticeable functions are attributed to this domain [125]. Previous studies have demonstrated that MAGE-A3 is involved in carcinogenesis via inhibition of apoptosis in tumor cells and the regulation of essential pathways associated with cell proliferation [126]. As a target antigen for cancer vaccines, MAGE-A3 has been applied in cancer vaccine development to test the possibility of cancer treatment in murine models. Due to the dominant position of CTLs in cancer therapy, scientists, first of all, focused on the HLA class I epitopes derived from MAGE-A3 and detected their capability in induction and activation of CTLs. Several CTL epitopes from MAGE-A3, restricted to HLA class I molecules such as HLA-A*1, HLA-A*2, HLA-A*24, and HLA-B*37, have been investigated [127,128,129,130]. These peptides exhibited potential antitumor functions in promoting CTL clones that recognize MAGE-A3-expressing tumors. With further understanding of Th-mediated immune response against malignant cells, increased attention was focused on Th lymphocytes’ critical role in the induction and maintenance of CTLs. Schultz et al. synthesized an HLA-DP4-restricted MAGE-A3 epitope [131]. After vaccination, CD4^+^ T cell clone directed against MAGE-A3 antigen and recognition of MAGE-A3-expressing tumor cells by CD4+ lymphocytes were observed. Furthermore, HLA-DR4- and HLA-DR7-restricted MAGE-A3 epitopes were also described by Kobayashi et al. [132]. These peptides induced specific T-cell response and enhanced recognition of tumor antigen by MAGE-A3-reactive Th clones. In addition to MAGE-A3 peptide vaccines, various other technology platforms have been utilized, such as DNA/RNA vaccines, DC-based vaccines, and viral vector/bacterial vector vaccines [133,134,135,136,137]. For instance, Liu et al. overexpressed MAGE-A3 and CALR in DCs via adenoviral transfection and used them as cancer vaccine to struggle against esophageal squamous cell carcinoma (ESCC) [135]. CALR/MAGE-A3-transfected DCs showed high expression of CD80, CD83, CD86 and were able to stimulate specific CD8^+^ CTLs targeted to ESCC cells expressing MAGE-A3. The construction of recombination fusion protein by linking MAGE-A3 peptides to adjuvant protein, or functional domain, is also a novel design strategy for MAGE-A3-based cancer vaccines. By fusion of MAGE-A3 peptides with cell-penetrating domains (CPDs), it was possible to achieve more efficient DC membrane penetration and induction of high-level expression of unique DC markers than with MAGE-A3 alone, making such fusion more potent therapeutic cancer vaccine compared with existing MAGE-A3 protein and peptide vaccines [138].

#### 3.1.2. “New York Esophageal Squamous Cell Carcinoma-1” Antigen

As an archetypal member of CGAs, NY-ESO-1 is encoded by the CTGAG1B gene located on the Xq28 region of the X chromosome [139]. The expression frequency of NY-ESO-1 varies significantly among different types of tumors. Most commonly expressing tumors are myxoid and round cell liposarcoma (89–100%), while other cancer types usually account for 20–40% [140,141,142,143]. In cancer patients, simultaneous humoral and cytotoxic responses against NY-ESO-1 were frequently observed, further revealing the natural ability of NY-ESO-1 to trigger a specific antitumor immune response [144,145]. Thus, there is a mainstream acceptance that NY-ESO-1 can serve as a promising candidate for cancer vaccines, and published results have supported this notion. Chen et al. synthesized HLA-A*2-restricted NY-ESO-1 peptide and used it to activate NY-ESO-1-specific immune response, resulting in NY-ESO-1-positive tumor cells killed by CTLs specific to correspondent peptide [146]. Zarour et al. identified HLA-DRB1*0401-presented epitopes and proved their capability to generate or enhance specific CD4+ T-cell responses against tumors expressing NY-ESO-1 in vivo [147]. In recent years, the form of NY-ESO-1-based DC, DNA, and oncolytic virus cancer vaccines have been designed, and favorable tumor inhibition results were observed [148,149,150]. Currently, effective adjuvant formulation, including aluminum salts (alum), CpG oligodeoxynucleotide (CpG), and innate defense regulator peptide HH2, combined with NY-ESO-1 antigen, was explored in murine tumor models by Yang’s group [151,152]. They showed that NY-ESO-1-alum-CpG-HH2 immunization elicited CTL response and increased TILs. This adjuvant strategy significantly upregulated the production of IFN-γ, TNF-α, and IL-1β, and enhanced uptake of antigen via activation of p38, Erk1/2, and NF-κB pathway.

#### 3.1.3. Preferentially Expressed Antigen in Melanoma

The PRAME gene is localized on chromosome 22 at locus 22q11.22. PRAME was first identified as a cancer antigen recognized by HLA-A*24 restricted CTLs in metastatic melanoma [153]. PRAME was found to be highly expressed in human adult germ cells and various tumor types. Meanwhile, limited expression of PRAME was also detected in ovaries, adrenals, and endometrium, making its expression germline-selective rather than typical germline-restricted [154]. As a novel diagnostic biomarker, an elevated PRAME expression level was correlated with more advanced malignant disease and a higher risk of metastasis, suggesting its pivotal role in cancer progression, including replicative immortality, invasion, and metastasis [155,156,157]. Thus, with further comprehension of unique features and biological mechanisms in tumorigenesis of PRAME, the candidate position of PRAME in cancer vaccines was probed to struggle against malignant diseases. Quintarelli et al. generated CTLs directed against HLA-A*02-restricted PRAME-peptides from healthy donors and chronic myelogenous leukemia (CML) patients. These CTLs recognized and had cytotoxic activity against PRAME-expressing tumor cell lines and primary CML cells, demonstrating the potential of PRAME in cancer vaccine development for CML treatment. Additionally, Rezvani et al. studied CD8^+^ T-cell responses to PRAME-derived epitopes in patients with acute lymphoblastic leukemia (ALL), acute myeloid leukemia (AML), and chronic myeloid leukemia (CML) [158]. Greater frequency of PRAME-specific CD8^+^ T cells was evident in patients with AML, CML, and ALL than healthy controls, revealing the potential for developing PRAME as a target for immunotherapy in leukemia. Furthermore, Gérard et al. tested the recombinant PRAME protein antitumor activity combined with AS15 immunostimulant in murine tumor models [159]. PRAME plus AS15 induced both CD4^+^ and CD8^+^ T-cell responses, as well as high PRAME-specific antibody titers, indicating that PRAME is potentially immunogenic in humans. Noteworthy, PRAME can bind retinoic acid (RA). RA is usually applied in hematologic malignancies to induce proliferation arrest, differentiation, and apoptosis. However, PRAME, binding to the retinoic acid receptors, prevents signing pathway activation and promotes tumorigenesis [160]. Thus, the possibility of a PRAME-based cancer vaccine to enhance the treatment response of all-trans retinoic acid (ATRA) treatment to ALL is under discussion [154].

### 3.2. Clinical Advance in Cancer-Germline Antigen-Based Vaccination

Immunogenicity and tumor specificity of CGAs have been tested in various preclinical studies, and results revealed a promising role of CGAs as prioritized targets for cancer vaccines. With these milestones, increasing scientific groups explored the efficacy of CGA-based vaccines in cancer treatment and their safety. These CGA-based vaccines were well-tolerated in cancer patients, observing specific T-cell responses, while clinical responses vary from trial to trial, as shown in Table S2. MAGE-A3-based cancer vaccines have been applied in multiple types of tumors in clinical trials, and results concerning their effectiveness and security were obtained. Two most comprehensive randomized, double-blinded, placebo-controlled phase III clinical trials using MAGE-A3 as target antigen in cancer vaccines, named MAGRIT and DERMA, were carried out for NSCLC and melanoma treatment, respectively [161,162]. In the MAGRIT trial, 13,849 patients were screened to select MAGE-A3-positive NSCLC patients, and 2312 of these patients were enrolled in this study. Patients were randomly assigned to receive 13 intramuscular injections of recombinant MAGE-A3 protein vaccine or placebo during 27 months [161]. Although this vaccine was well tolerated, and a low occurrence of vaccine-related serious adverse events was reported, unfortunately, this trial was stopped in 2014 due to the lack of changes in disease-free survival time in the MAGE-A3 immunotherapeutic group compared with the placebo group. In the other trial, DERMA, 895 MAGE-A3-positive melanoma patients received up to 13 intramuscular injections of recombinant MAGE-A3 with AS15 immunostimulant [162]. Final analysis revealed that median disease-free survival was 11 months in the MAGE-A3 group, while 11.2 months in the placebo group, demonstrating MAGE-A3 immunotherapeutic alone was not efficacious in melanoma treatment, even though rare treatment-related adverse events were reported. Therefore, this trial was terminated in 2016. Although the failure of these two phase III MAGE-A3-based cancer vaccine trials is discouraging, other formulations of MAGE-A3-based vaccines are still under investigation in other types of cancer. For instance, Krishnadas et al. applied MAGE-A1, MAGE-A3, and NY-ESO-1 peptides-pulsed DC vaccine in children with neuroblastoma or sarcoma in combination with decitabine for cancer treatment [163]. After vaccination, six out of nine patients experienced specific T-cell responses, whereas grade ≥3 adverse events occurred in five patients. According to the final analysis, one patient had a complete response while another one had a partial response. This combined strategy is feasible and immunogenic to some extent, but clinical benefit and toxicity still warrant further exploration.

Like MAGE-A3, NY-ESO-1 is also gaining significant attention in the field of cancer vaccines due to its specific immunogenic capability and aberrant expression in cancers. Based on NY-ESO-1, multiple types of cancer vaccines have been developed for tumor therapy. Phase I trial was carried out by Mahipal et al. to explore the efficacy of NY-ESO-1 recombinant protein vaccine, named G305, in solid tumors, including melanoma, ovarian carcinoma, breast carcinoma, etc. [164]. Six out of eleven patients developed specific CD4^+^ T-cell responses, whereas specific CD8^+^ T-cell activity was observed in four out of eleven patients, demonstrating immunogenicity of NY-ESO-1 in immune response initiation. Furthermore, three patients had a stable disease status one year after treatment, revealing potential clinical benefits for patients with solid tumors who will receive NY-ESO-1-based cancer vaccination. Meanwhile, a DC-based vaccine with DCs loaded with multiple TAAs, including NY-ESO-1, MAGE-C2, and MUC1, was designed by Westdorp’s group to apply in prostate carcinoma [165]. Twenty-one prostate carcinoma patients participated in this trial, and twelve of them experienced stable disease, while only one gained a partial clinical response, with no serious adverse events occurred among patients. Moreover, antigen-specific T cells were detected in peripheral blood in twelve out of twenty-one patients. This multiple TAAs-based vaccine was shown to be feasible and safe for potential clinical application, with an improved clinical outcome for prostate cancer patients. However, no phase III clinical trial results concerning NY-ESO-1-based cancer vaccines are currently available to our knowledge. Although one phase III, randomized, double-blind, placebo-controlled study of G305 vaccine application in sarcoma patients was started in 2018 (NCT03520959), this trial was terminated by a sponsor’s decision in 2020. Therefore, more phase III studies are earnestly needed to discuss the effectiveness of the NY-ESO-1-based vaccine in clinical application.

Several clinical trials related to PRAME have been reported and provided us bright insight into PRAME-based cancer vaccine development. Pujol et al. developed a recombinant PRAME protein vaccine for NSCLC patients to explore its safety and immunogenicity. In their phase I study, 26 out of 35 patients exploit specific CD4^+^ T- cell responses, while specific CD8^+^ T-cell responses only occurred in two patients [166]. No serious adverse events happened during administration. Another clinical trial reported by Weber et al. showed a peptide vaccine for solid tumor patients targeting PRAME and PSMA, a prostate-specific membrane antigen [167]. This vaccine induced a specific T-cell immunity toward tumors in 15 out of 24 patients, and ten patients achieved stable disease after two cycles of treatment. Although these two phase I studies revealed PRAME as a potential target in cancer vaccines, more data concerning overall survival and disease-free survival and further clinical trials would make PRAME a more convincing targeted molecule for cancer vaccine design.

### 3.3. Combination Therapy

Based on limited clinical benefits observed after vaccination, the application of CGA-based vaccines alone is still unsatisfying, especially the failure of two phase III MAGE-A3-based cancer vaccine trials for cancer treatment. Rational combination therapy may bolster immune response against tumors and gain more clinical benefits for cancer patients. Traditional cancer therapy, including chemotherapy and radiotherapy, has been found to work synergistically with immunotherapy by inducing increased antigens expression on the tumor cell surface or even releasing cancer antigens extracellularly [168]. Thus, theoretically, CGA-based vaccines combined with traditional therapeutics may increase the exposure of CGA on tumor cells and further recognition by T lymphocytes, contributing to the elimination of cancer cells. Furthermore, the major obstacle of chemotherapy application is drug resistance due to the anti-apoptotic capability that cancer cells exhibit, which is conceptually associated with cancer stem cells (CSCs). CGAs are involved in stem cell differentiation and carcinogenesis, demonstrating their unique role in CSCs, although the mechanism remains unclear [169]. Therefore, CGA-based cancer vaccines are an excellent choice to combine with chemotherapy by eliminating normal tumor cells and CSCs as a whole. Fukuda et al. employed this combined notion in melanoma treatment [170]. They developed a DC-based cancer vaccine with DCs loaded with MAGE-A3, MAGE-A2, and three other TAAs, combined with carboplatin and paclitaxel chemotherapy. On day one of a 28-day cycle, patients received intravenous carboplatin and paclitaxel administration, while on days eight and 22, intradermal injections of peptide-pulsed DC vaccine were performed. After three cycles, one of nine patients achieved a partial response, while four had stable disease. Median OS and PFS were 12 and 2.3 months, respectively. Their results provide the rationale for continued investigations of combination therapy comprising CGA-based cancer vaccines and chemotherapy.

ICBs block immunosuppression effects by targeting checkpoint molecules like PD-1 and CTLA-4, resulting in enhanced T cell activation and cytotoxicity against cancer cells. The use of ICBs was approved by the FDA to apply in melanoma and NSCLC and has dramatically improved cancer patients’ survival. Recently, combination approaches by using ICBs with other immunotherapies, especially cancer vaccines, have been proposed and practiced in clinical trials to circumvent limitations and enhance the effectiveness of individual immunotherapy approaches. Previous studies showed that PD-1 is highly expressed on CGA-specific tumor-infiltrating lymphocytes (TILs) and limits their antitumor function. After blocking the PD-1/PD-L1 pathway, the function and antitumor ability of MAGE-A3-specific CD8^+^ T cells were restored [171]. Similarly, NY-ESO-1-specific CD8^+^ T cells were negatively correlated with PD-1 expression in melanoma and ovarian cancer patients [172,173]. Moreover, blockade of CTLA-4 also enhances NY-ESO-1 specific T-cell responses in melanoma patients and bring clinical benefits [174]. In summary, all these findings provide a strong rationale to combine CGA-based cancer vaccines with ICBs for cancer treatment, and this combined strategy aim at eliminating malignant cells synergistically in a more comprehensive way. A preclinical study revealed that mice with melanoma, when vaccinated with a highly attenuated NY-ESO-1-expressing *Trypanosoma cruzi* strain, exhibit limited antitumor effects for preventing cancer development. However, when administrating CTLA-4 inhibitor during vaccination, a higher frequency of NY-ESO-1-specific effector CD8^+^ T cells producing IFN-γ was evident and more lymphocytes migrated and infiltrated the tumor microenvironment (TME), resulting in the prevention of tumor progression and extended survival of tumor-bearing mice [175]. Furthermore, this combined therapy also has been practiced in clinical trials. A study reported by Gibney et al. showed that 33 patients received a multi-peptide vaccine (gp100, MART-1, and NY-ESO-1) followed by nivolumab maintenance [176]. After vaccination, high PFS time (47.1 months) and an increase in tetramer-specific CD8^+^ T-cell populations were achieved. Thus, CGA-based vaccines should be considered in combination with ICBs for elevated antitumor effects.

The epigenetic regulation is critically involved in the expression of CGAs in normal and cancer tissues, while DNA methylation is one of the most important mechanisms underlying the high level of CGAs in tumors. DNA methyltransferase (DNMT) inhibitors, such as 5-aza-2′-deoxycytidine (5-aza-2′-CdR) or 5-azacytidine, have been approved for cancer treatment by reversing epigenetic silencing of genes to enhance the immunogenic potential of cancer cells [177,178]. When applying DNMT inhibitors in cancer patients, the upregulated expression of CGAs was frequently observed with more active spontaneous immunity against CGAs [179]. Therefore, a combination of CGA-based vaccines and DNMT inhibitors will induce more CGAs expression in tumors and trigger highly activated CGA-specific T-cell responses. The feasibility of this combined strategy has already been tested by Odunsi et al. [180]. They performed a phase I dose-escalation trial of decitabine (5-aza-20-deoxycytidine) as an addition to the NY-ESO-1-based vaccine in ovarian carcinoma patients. This combined strategy benefits most patients from increased NY-ESO-1 serum antibodies and T-cell responses while with limited toxicities. In the final clinical evaluation, out of ten patients, five had stable disease, and one had a partial response. Based on these encouraging results, a combination of CGA-based vaccine and DNMT inhibitors warrants further exploration in other types of cancer. However, the elevated expression of CGAs in multiple tumors is usually associated with tumor progression and aggressive type. Thus, to avoid latent deleterious effects on patients, the choice and usage of DNMT inhibitors, which could potentially induce CGAs expression in tumors, need to be cautious when employed together with CGA-based cancer vaccines.

## 4. Antigen Selection Strategy for Cancer Vaccine

Although some cancer antigens have already shown their advantage in cancer vaccine development, none of them are entirely suitable for every kind of cancer, even for different individuals with the same disease. To draw a better cancer antigen selection strategy for cancer vaccines, comparison among cancer antigens, and knowing their advantages and disadvantages, is essential. Hereby, we summarized profits and defects that CGAs, neoantigens, and viral antigens will bring for cancer vaccine design (Table 2).

Immune-privileged antigens have been proven to be expressed in various kinds of cancers, and based on this fact, cancer vaccines that target immune-privileged antigens can be applied in a broad spectrum of malignant diseases. The feature of a wide range of adaptability endows immune-privileged antigen-based vaccines a good clinical translation. The whole gene and amino acid sequence of most immune-privileged antigens have been well-established; thus, immune-privileged antigen-based vaccines can be generated through established manufacturing technologies in mass production. Therefore, when a patient is diagnosed to bear immune-privileged antigen-expressing tumor, immune-privileged antigen-based vaccines are already on stand by and can be administered to the patient in time, avoiding time consumption in manufacturing and disease progression during vaccine production. Furthermore, with established production technologies and simple diagnostic methods, immune-privileged antigen-based vaccines are more affordable for cancer patients. However, except for immune-privileged tissues, not all immune-privileged antigens are absent from normal tissues. For instance, although PRAME exists mainly in germ cells, limited PRAME expression is also evident in ovaries, adrenals, and endometrium [154]. Thus, theoretically, the PRAME-based cancer vaccine has a risk of autoimmune response induction against normal tissues. Another type of cancer antigen—cancer-retina antigens—has been recently described [181]. Found to be expressed in a number of malignancies and retina, thus being highly immunogenic due to their immune-privileged status, they are considered to be immunogens for cancer vaccines besides CGAs [182,183]. Preclinical studies for analyzing the possibility of cancer-retina antigens induce tumor-specific immune response are required to be undertaken. However, although no cases concerning CGA-based vaccine-related autoimmune testopathy were reported, theoretically, cancer-retina antigen-based vaccines might induce an autoimmune response that will lead to the degradation of the retina. This statement is supported by reported cases of paraneoplastic syndromes of tumor-related retinopathy when antibodies against retinal proteins were produced in response to cancer-retina antigen-producing tumor and were responsible for retina degradation [184]. Thus, possible autoimmunity induced by such vaccines should be considered during vaccine composition preparation, e.g., by reducing HLA class II-specific epitopes. Additionally, tumor heterogeneity is also one obstacle that threatens the efficacy of immune-privileged antigen-based cancer vaccines. It does not make sense to administrate such immune-privileged antigen-based vaccines if the patient’s tumor does not express the correspondent antigen.

**Figure 4:**
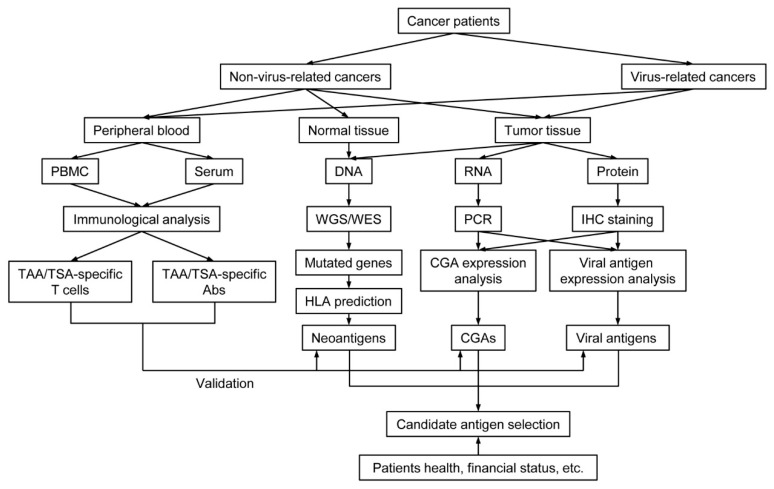
Methodology pipeline for antigen selection in cancer vaccine production. For patients with non-virus-related cancers, tumor biopsy samples and corresponding normal tissues are obtained and conducted to WGS/WES analysis for mutated genes. By using bioinformatic algorithms, neoantigens with high HLA binding affinity are selected from mutated proteins as candidate antigens for cancer vaccine design. Meanwhile, the expression of CGAs is determined by PCR and IHC (immunohistochemistry) staining methods in tumor tissues. CGAs with highly elevated levels are considered as candidates for vaccines. As for virus-related cancer patients, the first consideration for vaccine development is viral antigens. PCR and IHC staining are performed to evaluate the expression of viral antigens in the tumor biopsy. Based on the analysis results, elevated viral antigens are selected as candidate antigens for cancer vaccine manufacturing. Furthermore, immunological experiments should be carried out to access the specific T cells and antibodies against candidate antigens by using the peripheral blood sample from patients, validating the potential role of selected candidate antigens. After balancing patients’ health situation and financial status, a final decision concerning antigens for cancer vaccine development is made. PBMC—peripheral blood mononuclear cells.

By targeting proteins derived from mutated genes in tumors, neoantigen-based vaccines show a robust capability to induce highly specific antitumor immunity in patients. These mutated proteins are foreign to the immune system, possessing low central tolerance. When neoantigen-based vaccines are recognized by the immune system, a robust and specific immunity will be triggered against them and neoantigen-expressing tumors, leading to their elimination eventually. Neoantigen-based vaccines accurately target tumors, providing cancer patients a personalized immune strategy with high specificity. This vaccine meets the criteria of the current trend of precision medicine in cancer treatment. By sequencing the genome of tumor tissues and compared with the genome of normal tissues, multiple neoepitopes can be selected for vaccine manufacturing to eliminate cancer cells comprehensively and overcome tumor heterogeneity to the greatest extent possible. However, complex preparation procedures and immature manufacturing technologies contribute to the difficulty of neoantigen-based vaccine development (Figure 4). Moreover, although next-generation sequencing achieved a quick progression recently, the cost of WGS/WES is still unaffordable for most patients, making neoantigen-based vaccine hard to be applied universally. Moreover, time consumption also threatens the position of the neoantigen-based vaccines in immunotherapy. It usually takes 2–3 months to design and prepare a neoantigen-based vaccine for cancer patients. Thereafter, most cancer patients develop disease progression during the waiting period for neoantigen-based vaccine manufacturing, making them not more suitable for vaccination.

As one principal member of TSAs, viral antigens also possess high specific immunogenicity and low central tolerance, making them probably an ideal target for cancer vaccines. After recognition and procession by the immune system, viral antigen-based vaccines can trigger a substantial and specific immune response against virus-related tumors. With available knowledge of virus etiology, the manufacture of viral antigen-based vaccines is not difficult to establish (Figure 4). Moreover, established prophylactic viral vaccine manufacturing technologies can be used as a reference when constructing therapeutic viral antigen-based vaccines. However, the application of viral antigen-based vaccines is only suitable for virus-related cancers, whereas cancers without viral etiology hard to gain benefits from it. Furthermore, mutation frequently occurs in pathogenic viruses, which may change the amino acid sequence of targeted viral proteins, leading to loss of viral antigen-based vaccine immunogenicity.

**Figure 5:**
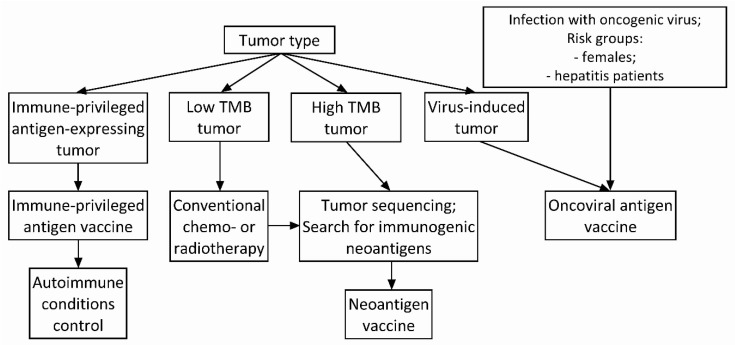
Tumor antigen selection strategy for cancer vaccines.

Objectively, there is not enough clinical trial data to conclude which antigen or group of antigens is more favorable to choose. Pros and cons of different tumor antigen groups can be highlighted, but what is out of any discussions is that there is no single antigen or a single group of them, which will be applied for every case of cancer type. It is obvious and already can be traced from little available clinical trial data that utilizing the same antigen for different malignancies treatment results in different antigen-containing vaccine efficacy. It happens because malignant diseases are very heterogeneous from type to type. Moreover, they are heterogeneous from individual to individual. Thus, the current trend in modern medicine, including oncology, is establishing personalized diagnostics and treatment approaches. It is required to develop conformable approaches for cancer treatment selection in an individual case. It means that specific patient’s parameters should be considered in order to form a picture of the choice of one or another approach to treatment. For the selection of cancer vaccines, it is essential to consider the patient’s tumor antigens to select appropriate vaccine composition and a platform for antigen delivery [185,186]. Pros and cons should then be turned into conditions, which will help understand where to use this or that antigen (Figure 5). Even a combination of antigens from different groups can be applied to enhance vaccine efficacy if there are appropriate conditions (e.g., virus-induced malignancy with high TMB). To summarize, there should be a panel of different available antigens that should be adapted individually, rather than utilizing a single group of antigens for each patient. Therefore, exploratory research of tumor antigens and their identification approaches are required to cover possible tumor antigenic variations.

## Figures and Tables

**Table 1:** Clinical trials of neoantigen-encoded cancer vaccines.

Type of Intervention	Phase	Status as of October 2020	Disease	Starting Date	Trial ID
Peptides	I	Recruiting	Pancrease carcinoma	April 2018	NCT03558945
Peptides	I	Recruiting	NSCLC	May 2020	NCT04487093
Peptides	Ⅰ	Not yet recruiting	Melanoma	September 2020	NCT03929029
Peptides	Ⅰ	Recruiting	NSCLC	May 2020	NCT04397926
Peptides	Ⅱ	Recruiting	Breast carcinoma	December 2018	NCT03606967
Peptides	Ⅰ	Recruiting	Renal carcinoma	March 2019	NCT02950766
Peptides	Ⅰ	Recruiting	Urothelial carcinoma	May 2019	NCT03359239
Peptides	Ⅰ	Not yet recruiting	Lymphocytic leukemia	September 2020	NCT03219450
DC	Ⅰ	Recruiting	Hepatocellular carcinoma	October 2018	NCT03674073
DC	I/II	Active	Colorectal carcinoma	October 2010	NCT01885702
DC	Ⅰ	Recruiting	NSCLC	August 2019	NCT04078269
DC	I/II	Unknown	Biliary tract carcinoma	September 2015	NCT02632019
DNA	Ⅰ	Recruiting	Breast carcinoma	August 2019	NCT03199040
DNA	Ⅰ	Active	Pancrease carcinoma	January 2018	NCT03122106
DNA	Ⅱ	Not yet recruiting	NSCLC	January 2021	NCT04397003
DNA	Ⅰ	Recruiting	Glioblastoma	July 2020	NCT04015700
RNA	Ⅰ/Ⅱ	Terminated	Solid tumors	May 2018	NCT03480152
RNA	N/A	Not yet recruiting	Esophageal carcinoma, NSCLC	May 2019	NCT03908671
RNA	N/A	Recruiting	Solid tumors	May 2018	NCT03468244

N/A—not applicable; NSCLC—non-small-cell lung carcinoma.

**Table 2:** Pros and cons of different tumor antigens in vaccine design.

Cancer Antigen Type	Pros	Cons
Immune-privileged antigens	A wide range of adaptability Well-established manufacturing technologies Simple preparation procedures Time- and cost-effective Relatively high specificity6. Low central tolerance Low central tolerance	Potential autoimmune responses Limited effects due to tumor heterogeneity
Neoantigens	High specificity Low central tolerance Personalized strategy for patients High affinity to HLA molecules and TCRs	Great money and time consumption Complex preparation procedures Immature preparation technologies
Viral antigens	Target virus-related cancers specifically Low central tolerance Simple preparation procedures Well-established manufacturing technologies	Restricted to virus-related cancers Loss of immunogenicity due to mutations in viruses

## Data Availability

Data available in a publicly accessible repository.

## Supplementary Materials

The following are available online at https://www.mdpi.com/2076-393X/9/2/85/s1, Table S1. Selective clinical trials of viral antigen-based cancer vaccines; Table S2. Selective clinical trials of CGAs-based cancer vaccines.

## References

[1] Lee M.Y., Jeon J.W., Sievers C., Allen C.T. (2020). Antigen processing and presentation in cancer immunotherapy. J. Immunother. Cancer.

[2] Yarchoan M., Johnson B.A., Lutz E.R., Laheru D.A., Jaffee E.M. (2017). Targeting neoantigens to augment antitumour immunity. Nat. Rev. Cancer.

[3] Smith C.C., Selitsky S.R., Chai S., Armistead P.M., Vincent B.G., Serody J.S. (2019). Alternative tumour-specific antigens. Nat. Rev. Cancer.

[4] Garraway L.A., Lander E.S. (2013). Lessons from the cancer genome. Cell.

[5] Segal N.H., Parsons D.W., Peggs K.S., Velculescu V., Kinzler K.W., Vogelstein B., Allison J.P. (2008). Epitope landscape in breast and colorectal cancer. Cancer Res..

[6] Wood L.D., Parsons D.W., Jones S., Lin J., Sjöblom T., Leary R.J., Shen D., Boca S.M., Barber T., Ptak J. (2007). The genomic landscapes of human breast and colorectal cancers. Science.

[7] De Plaen E., Lurquin C., Van Pel A., Mariamé B., Szikora J.P., Wölfel T., Sibille C., Chomez P., Boon T. (1988). Immunogenic (tum-) variants of mouse tumor P815: Cloning of the gene of tum- antigen P91A and identification of the tum- mutation. Proc. Natl. Acad. Sci. USA.

[8] Sibille C., Chomez P., Wildmann C., Van Pel A., De Plaen E., Maryanski J.L., de Bergeyck V., Boon T. (1990). Structure of the gene of tum- transplantation antigen P198: A point mutation generates a new antigenic peptide. J. Exp. Med..

[9] Monach P.A., Meredith S.C., Siegel C.T., Schreiber H. (1995). A unique tumor antigen produced by a single amino acid substitution. Immunity.

[10] Coulie P.G., Lehmann F., Lethe B., Herman J., Lurquin C., Andrawiss M., Boon T. (1995). A mutated intron sequence codes for an antigenic peptide recognized by cytolytic T lymphocytes on a human melanoma. Proc. Natl. Acad. Sci. USA.

[11] Wolfel T., Hauer M., Schneider J., Serrano M., Wolfel C., Klehmann-Hieb E., De Plaen E., Hankeln T., Meyer zum Buschenfelde K.H., Beach D. (1995). A p16INK4a-insensitive CDK4 mutant targeted by cytolytic T lymphocytes in a human melanoma. Science.

[12] Chen D.S., Mellman I. (2017). Elements of cancer immunity and the cancer-immune set point. Nature.

[13] Besser H., Yunger S., Merhavi-Shoham E., Cohen C.J., Louzoun Y. (2019). Level of neo-epitope predecessor and mutation type determine T cell activation of MHC binding peptides. J. Immunother. Cancer.

[14] Nielsen J.S., Chang A.R., Wick D.A., Sedgwick C.G., Zong Z., Mungall A.J., Martin S.D., Kinloch N.N., Ott-Langer S., Brumme Z.L. (2017). Mapping the human T cell repertoire to recurrent driver mutations in MYD88 and EZH2 in lymphoma. Oncoimmunology.

[15] Aleksic M., Liddy N., Molloy P.E., Pumphrey N., Vuidepot A., Chang K.M., Jakobsen B.K. (2012). Different affinity windows for virus and cancer-specific T-cell receptors: Implications for therapeutic strategies. Eur. J. Immunol..

[16] Stone J.D., Harris D.T., Kranz D.M. (2015). TCR affinity for p/MHC formed by tumor antigens that are self-proteins: Impact on efficacy and toxicity. Curr. Opin. Immunol..

[17] Xu P., Luo H., Kong Y., Lai W.F., Cui L., Zhu X. (2020). Cancer neoantigen: Boosting immunotherapy. Biomed. Pharmacother..

[18] Karpanen T., Olweus J. (2017). The Potential of Donor T-Cell Repertoires in Neoantigen-Targeted Cancer Immunotherapy. Front. Immunol..

[19] Van Buuren M.M., Calis J.J., Schumacher T.N. (2014). High sensitivity of cancer exome-based CD8 T cell neo-antigen identification. Oncoimmunology.

[20] Gopanenko A.V., Kosobokova E.N., Kosorukov V.S. (2020). Main Strategies for the Identification of Neoantigens. Cancers.

[21] Nakagawa H., Fujita M. (2018). Whole genome sequencing analysis for cancer genomics and precision medicine. Cancer Sci..

[22] Chen F., Zou Z., Du J., Su S., Shao J., Meng F., Yang J., Xu Q., Ding N., Yang Y. (2019). Neoantigen identification strategies enable personalized immunotherapy in refractory solid tumors. J. Clin. Investig..

[23] Sneddon S., Rive C.M., Ma S., Dick I.M., Allcock R.J.N., Brown S.D., Holt R.A., Watson M., Leary S., Lee Y.C.G. (2020). Identification of a CD8+ T-cell response to a predicted neoantigen in malignant mesothelioma. Oncoimmunology.

[24] Hos B.J., Camps M.G.M., van den Bulk J., Tondini E., van den Ende T.C., Ruano D., Franken K., Janssen G.M.C., Ru A., Filippov D.V. (2019). Identification of a neo-epitope dominating endogenous CD8 T cell responses to MC-38 colorectal cancer. Oncoimmunology.

[25] Bjerregaard A.M., Nielsen M., Hadrup S.R., Szallasi Z., Eklund A.C. (2017). MuPeXI: Prediction of neo-epitopes from tumor sequencing data. Cancer Immunol. Immunother. CII.

[26] Hundal J., Carreno B.M., Petti A.A., Linette G.P., Griffith O.L., Mardis E.R., Griffith M. (2016). pVAC-Seq: A genome-guided in silico approach to identifying tumor neoantigens. Genome Med..

[27] Szolek A., Schubert B., Mohr C., Sturm M., Feldhahn M., Kohlbacher O. (2014). OptiType: Precision HLA typing from next-generation sequencing data. Bioinformatics.

[28] Bais P., Namburi S., Gatti D.M., Zhang X., Chuang J.H. (2017). CloudNeo: A cloud pipeline for identifying patient-specific tumor neoantigens. Bioinformatics.

[29] Schenck R.O., Lakatos E., Gatenbee C., Graham T.A., Anderson A.R.A. (2019). NeoPredPipe: High-throughput neoantigen prediction and recognition potential pipeline. BMC Bioinform..

[30] Chen R., Fulton K.M., Twine S.M., Li J. (2019). Identification of mhc peptides using mass spectrometry for neoantigen discovery and cancer vaccine developmeNT. Mass Spectrom. Rev..

[31] Zhang X., Qi Y., Zhang Q., Liu W. (2019). Application of mass spectrometry-based MHC immunopeptidome profiling in neoantigen identification for tumor immunotherapy. Biomed. Pharmacother..

[32] Li L., Goedegebuure S.P., Gillanders W.E. (2017). Preclinical and clinical development of neoantigen vaccines. Ann. Oncol..

[33] Chen X., Yang J., Wang L., Liu B. (2020). Personalized neoantigen vaccination with synthetic long peptides: Recent advances and future perspectives. Theranostics.

[34] Ott P.A., Hu Z., Keskin D.B., Shukla S.A., Sun J., Bozym D.J., Zhang W., Luoma A., Giobbie-Hurder A., Peter L. (2017). An immunogenic personal neoantigen vaccine for patients with melanoma. Nature.

[35] Carreno B.M., Magrini V., Becker-Hapak M., Kaabinejadian S., Hundal J., Petti A.A., Ly A., Lie W.R., Hildebrand W.H., Mardis E.R. (2015). Cancer immunotherapy. A dendritic cell vaccine increases the breadth and diversity of melanoma neoantigen-specific T cells. Science.

[36] Morisaki T., Hikichi T., Onishi H., Morisaki T., Kubo M., Hirano T., Yoshimura S., Kiyotani K., Nakamura Y. (2020). Intranodal Administration of Neoantigen Peptide-loaded Dendritic Cell Vaccine Elicits Epitope-specific T Cell Responses and Clinical Effects in a Patient with Chemorefractory Ovarian Cancer with Malignant Ascites. Immunol. Investig..

[37] Zhang R., Yuan F., Shu Y., Tian Y., Zhou B., Yi L., Zhang X., Ding Z., Xu H., Yang L. (2020). Personalized neoantigen-pulsed dendritic cell vaccines show superior immunogenicity to neoantigen-adjuvant vaccines in mouse tumor models. Cancer Immunol. Immunother. CII.

[38] Duperret E.K., Perales-Puchalt A., Stoltz R., Hiranjith G.H., Mandloi N., Barlow J., Chaudhuri A., Sardesai N.Y., Weiner D.B. (2019). A Synthetic DNA, Multi-Neoantigen Vaccine Drives Predominately MHC Class I CD8(+) T-cell Responses, Impacting Tumor Challenge. Cancer Immunol. Res..

[39] Sahin U., Derhovanessian E., Miller M., Kloke B.P., Simon P., Lower M., Bukur V., Tadmor A.D., Luxemburger U., Schrors B. (2017). Personalized RNA mutanome vaccines mobilize poly-specific therapeutic immunity against cancer. Nature.

[40] Salomon N., Vascotto F., Selmi A., Vormehr M., Quinkhardt J., Bukur T., Schrörs B., Löewer M., Diken M., Türeci Ö. (2020). A liposomal RNA vaccine inducing neoantigen-specific CD4(+) T cells augments the antitumor activity of local radiotherapy in mice. Oncoimmunology.

[41] Zhu G., Zhang F., Ni Q., Niu G., Chen X. (2017). Efficient Nanovaccine Delivery in Cancer Immunotherapy. ACS Nano.

[42] Reuven E.M., Leviatan Ben-Arye S., Yu H., Duchi R., Perota A., Conchon S., Bachar Abramovitch S., Soulillou J.P., Galli C., Chen X. (2019). Biomimetic Glyconanoparticle Vaccine for Cancer Immunotherapy. ACS Nano.

[43] Mohsen M.O., Vogel M., Riether C., Muller J., Salatino S., Ternette N., Gomes A.C., Cabral-Miranda G., El-Turabi A., Ruedl C. (2019). Targeting Mutated Plus Germline Epitopes Confers Pre-clinical Efficacy of an Instantly Formulated Cancer Nano-Vaccine. Front. Immunol..

[44] Aurisicchio L., Salvatori E., Lione L., Bandini S., Pallocca M., Maggio R., Fanciulli M., De Nicola F., Goeman F., Ciliberto G. (2019). Poly-specific neoantigen-targeted cancer vaccines delay patient derived tumor growth. J. Exp. Clin. Cancer Res..

[45] Kreiter S., Vormehr M., van de Roemer N., Diken M., Löwer M., Diekmann J., Boegel S., Schrörs B., Vascotto F., Castle J.C. (2015). Mutant MHC class II epitopes drive therapeutic immune responses to cancer. Nature.

[46] Zhang Y., Lin Z., Wan Y., Cai H., Deng L., Li R. (2019). The Immunogenicity and Anti-tumor Efficacy of a Rationally Designed Neoantigen Vaccine for B16F10 Mouse Melanoma. Front. Immunol..

[47] Scheetz L., Kadiyala P., Sun X., Son S., Hassani Najafabadi A., Aikins M., Lowenstein P.R., Schwendeman A., Castro M.G., Moon J.J. (2020). Synthetic High-density Lipoprotein Nanodiscs for Personalized Immunotherapy Against Gliomas. Clin. Cancer Res..

[48] Schumacher T., Bunse L., Pusch S., Sahm F., Wiestler B., Quandt J., Menn O., Osswald M., Oezen I., Ott M. (2014). A vaccine targeting mutant IDH1 induces antitumour immunity. Nature.

[49] Kinkead H.L., Hopkins A., Lutz E., Wu A.A., Yarchoan M., Cruz K., Woolman S., Vithayathil T., Glickman L.H., Ndubaku C.O. (2018). Combining STING-based neoantigen-targeted vaccine with checkpoint modulators enhances antitumor immunity in murine pancreatic cancer. JCI Insight.

[50] Forghanifard M.M., Gholamin M., Moaven O., Farshchian M., Ghahraman M., Aledavood A., Abbaszadegan M.R. (2014). Neoantigen in esophageal squamous cell carcinoma for dendritic cell-based cancer vaccine development. Med. Oncol..

[51] Keskin D.B., Anandappa A.J., Sun J., Tirosh I., Mathewson N.D., Li S., Oliveira G., Giobbie-Hurder A., Felt K., Gjini E. (2019). Neoantigen vaccine generates intratumoral T cell responses in phase Ib glioblastoma trial. Nature.

[52] Hilf N., Kuttruff-Coqui S., Frenzel K., Bukur V., Stevanovic S., Gouttefangeas C., Platten M., Tabatabai G., Dutoit V., van der Burg S.H. (2019). Actively personalized vaccination trial for newly diagnosed glioblastoma. Nature.

[53] Yadav M., Jhunjhunwala S., Phung Q.T., Lupardus P., Tanguay J., Bumbaca S., Franci C., Cheung T.K., Fritsche J., Weinschenk T. (2014). Predicting immunogenic tumour mutations by combining mass spectrometry and exome sequencing. Nature.

[54] McGranahan N., Furness A.J., Rosenthal R., Ramskov S., Lyngaa R., Saini S.K., Jamal-Hanjani M., Wilson G.A., Birkbak N.J., Hiley C.T. (2016). Clonal neoantigens elicit T cell immunoreactivity and sensitivity to immune checkpoint blockade. Science.

[55] Cook A.M., Lesterhuis W.J., Nowak A.K., Lake R.A. (2016). Chemotherapy and immunotherapy: Mapping the road ahead. Curr. Opin. Immunol..

[56] Gotwals P., Cameron S., Cipolletta D., Cremasco V., Crystal A., Hewes B., Mueller B., Quaratino S., Sabatos-Peyton C., Petruzzelli L. (2017). Prospects for combining targeted and conventional cancer therapy with immunotherapy. Nat. Rev. Cancer.

[57] Wu J., Waxman D.J. (2018). Immunogenic chemotherapy: Dose and schedule dependence and combination with immunotherapy. Cancer Lett..

[58] Boccardo E., Villa L.L. (2007). Viral origins of human cancer. Curr. Med. Chem..

[59] Coffin J.M., Hughes S.H., Varmus H.E. (1997). Retroviruses. Retroviruses.

[60] Valdespino V., Gorodezky C., Ortiz V., Kaufmann A.M., Roman-Basaure E., Vazquez A., Berumen J. (2005). HPV16-specific cytotoxic T lymphocyte responses are detected in all HPV16-positive cervical cancer patients. Gynecol. Oncol..

[61] Drury S.E., Gough R.E., McArthur S., Jessop M. (1998). Detection of herpesvirus-like and papillomavirus-like particles associated with diseases of tortoises. Vet. Rec..

[62] Mühr L.S.A., Eklund C., Dillner J. (2018). Towards quality and order in human papillomavirus research. Virology.

[63] Bosch F.X., Lorincz A., Muñoz N., Meijer C.J., Shah K.V. (2002). The causal relation between human papillomavirus and cervical cancer. J. Clin. Pathol..

[64] D’Souza G., Kreimer A.R., Viscidi R., Pawlita M., Fakhry C., Koch W.M., Westra W.H., Gillison M.L. (2007). Case-control study of human papillomavirus and oropharyngeal cancer. N. Engl. J. Med..

[65] Wang R., Pan W., Jin L., Huang W., Li Y., Wu D., Gao C., Ma D., Liao S. (2020). Human papillomavirus vaccine against cervical cancer: Opportunity and challenge. Cancer Lett..

[66] Scheffner M., Huibregtse J.M., Vierstra R.D., Howley P.M. (1993). The HPV-16 E6 and E6-AP complex functions as a ubiquitin-protein ligase in the ubiquitination of p53. Cell.

[67] Dyson N., Howley P.M., Münger K., Harlow E. (1989). The human papilloma virus-16 E7 oncoprotein is able to bind to the retinoblastoma gene product. Science.

[68] Van der Burg S.H., Melief C.J. (2011). Therapeutic vaccination against human papilloma virus induced malignancies. Curr. Opin. Immunol..

[69] Morrow M.P., Yan J., Sardesai N.Y. (2013). Human papillomavirus therapeutic vaccines: Targeting viral antigens as immunotherapy for precancerous disease and cancer. Expert Rev. Vaccines.

[70] Monnier-Benoit S., Mauny F., Riethmuller D., Guerrini J.S., Căpîlna M., Félix S., Seillès E., Mougin C., Prétet J.L. (2006). Immunohistochemical analysis of CD4+ and CD8+ T-cell subsets in high risk human papillomavirus-associated pre-malignant and malignant lesions of the uterine cervix. Gynecol. Oncol..

[71] Kim K.H., Greenfield W.W., Cannon M.J., Coleman H.N., Spencer H.J., Nakagawa M. (2012). CD4+ T-cell response against human papillomavirus type 16 E6 protein is associated with a favorable clinical trend. Cancer Immunol. Immunother. CII.

[72] Kim T.J., Jin H.T., Hur S.Y., Yang H.G., Seo Y.B., Hong S.R., Lee C.W., Kim S., Woo J.W., Park K.S. (2014). Clearance of persistent HPV infection and cervical lesion by therapeutic DNA vaccine in CIN3 patients. Nat. Commun..

[73] Khan S., Oosterhuis K., Wunderlich K., Bunnik E.M., Bhaggoe M., Boedhoe S., Karia S., Steenbergen R.D.M., Bosch L., Serroyen J. (2017). Development of a replication-deficient adenoviral vector-based vaccine candidate for the interception of HPV16- and HPV18-induced infections and disease. Int. J. cancer.

[74] Wallecha A., French C., Petit R., Singh R., Amin A., Rothman J. (2012). Lm-LLO-Based Immunotherapies and HPV-Associated Disease. J. Oncol..

[75] Rahma O.E., Herrin V.E., Ibrahim R.A., Toubaji A., Bernstein S., Dakheel O., Steinberg S.M., Abu Eid R., Mkrtichyan M., Berzofsky J.A. (2014). Pre-immature dendritic cells (PIDC) pulsed with HPV16 E6 or E7 peptide are capable of eliciting specific immune response in patients with advanced cervical cancer. J. Transl. Med..

[76] Kenter G.G., Welters M.J., Valentijn A.R., Lowik M.J., Berends-van der Meer D.M., Vloon A.P., Essahsah F., Fathers L.M., Offringa R., Drijfhout J.W. (2009). Vaccination against HPV-16 oncoproteins for vulvar intraepithelial neoplasia. N. Engl. J. Med..

[77] Stöppler M.C., Straight S.W., Tsao G., Schlegel R., McCance D.J. (1996). The E5 gene of HPV-16 enhances keratinocyte immortalization by full-length DNA. Virology.

[78] Straight S.W., Herman B., McCance D.J. (1995). The E5 oncoprotein of human papillomavirus type 16 inhibits the acidification of endosomes in human keratinocytes. J. Virol..

[79] Kim M.K., Kim H.S., Kim S.H., Oh J.M., Han J.Y., Lim J.M., Juhnn Y.S., Song Y.S. (2010). Human papillomavirus type 16 E5 oncoprotein as a new target for cervical cancer treatment. Biochem. Pharmacol..

[80] Liao S.J., Deng D.R., Zeng D., Zhang L., Hu X.J., Zhang W.N., Li L., Jiang X.F., Wang C.Y., Zhou J.F. (2013). HPV16 E5 peptide vaccine in treatment of cervical cancer in vitro and in vivo. J. Huazhong Univ. Sci. Technol. Med. Sci..

[81] Yuen M.F., Chen D.S., Dusheiko G.M., Janssen H.L.A., Lau D.T.Y., Locarnini S.A., Peters M.G., Lai C.L. (2018). Hepatitis B virus infection. Nat. Rev. Dis. Primers.

[82] Xu H.Z., Liu Y.P., Guleng B., Ren J.L. (2014). Hepatitis B Virus-Related Hepatocellular Carcinoma: Pathogenic Mechanisms and Novel Therapeutic Interventions. Gastrointest. Tumors.

[83] Tu W., Gong J., Tian D., Wang Z. (2019). Hepatitis B Virus X Protein Induces SATB1 Expression Through Activation of ERK and p38MAPK Pathways to Suppress Anoikis. Dig. Dis. Sci..

[84] Chen M.J., Wu D.W., Shen C.J., Cheng Y.M., Wu C.C., Lee H. (2020). Hepatitis B virus X protein promotes tumor invasion and poor prognosis in hepatocellular carcinoma via phosphorylation of paxillin at Serine 178 by activation of the c-Jun NH2-terminal kinase. Am. J. Cancer Res..

[85] Lupberger J., Hildt E. (2007). Hepatitis B virus-induced oncogenesis. World J. Gastroenterol..

[86] Ding F.X., Wang F., Lu Y.M., Li K., Wang K.H., He X.W., Sun S.H. (2009). Multiepitope peptide-loaded virus-like particles as a vaccine against hepatitis B virus-related hepatocellular carcinoma. Hepatology.

[87] Chen Y., Yang D., Li S., Gao Y., Jiang R., Deng L., Frankel F.R., Sun B. (2012). Development of a Listeria monocytogenes-based vaccine against hepatocellular carcinoma. Oncogene.

[88] Rongrui L., Na H., Zongfang L., Fanpu J., Shiwen J. (2014). Epigenetic mechanism involved in the HBV/HCV-related hepatocellular carcinoma tumorigenesis. Curr. Pharm. Des..

[89] Navas M.C., Glaser S., Dhruv H., Celinski S., Alpini G., Meng F. (2019). Hepatitis C Virus Infection and Cholangiocarcinoma: An Insight into Epidemiologic Evidences and Hypothetical Mechanisms of Oncogenesis. Am. J. Pathol..

[90] Preciado M.V., Valva P., Escobar-Gutierrez A., Rahal P., Ruiz-Tovar K., Yamasaki L., Vazquez-Chacon C., Martinez-Guarneros A., Carpio-Pedroza J.C., Fonseca-Coronado S. (2014). Hepatitis C virus molecular evolution: Transmission, disease progression and antiviral therapy. World J. Gastroenterol..

[91] Tagliamonte M., Petrizzo A., Napolitano M., Luciano A., Arra C., Maiolino P., Izzo F., Tornesello M.L., Aurisicchio L., Ciliberto G. (2015). Novel metronomic chemotherapy and cancer vaccine combinatorial strategy for hepatocellular carcinoma in a mouse model. Cancer Immunol. Immunother. CII.

[92] Nowalk A., Green M. (2016). Epstein-Barr Virus. Microbiol. Spectr..

[93] Teow S.Y., Yap H.Y., Peh S.C. (2017). Epstein-Barr Virus as a Promising Immunotherapeutic Target for Nasopharyngeal Carcinoma Treatment. J. Pathog.

[94] Duraiswamy J., Sherritt M., Thomson S., Tellam J., Cooper L., Connolly G., Bharadwaj M., Khanna R. (2003). Therapeutic LMP1 polyepitope vaccine for EBV-associated Hodgkin disease and nasopharyngeal carcinoma. Blood.

[95] Pan J., Zhang Q., Zhou J., Ma D., Xiao X., Wang D.W. (2009). Recombinant adeno-associated virus encoding Epstein-Barr virus latent membrane proteins fused with heat shock protein as a potential vaccine for nasopharyngeal carcinoma. Mol. Cancer Ther..

[96] Taylor G.S., Haigh T.A., Gudgeon N.H., Phelps R.J., Lee S.P., Steven N.M., Rickinson A.B. (2004). Dual stimulation of Epstein-Barr Virus (EBV)-specific CD4^+^- and CD8^+^-T-cell responses by a chimeric antigen construct: Potential therapeutic vaccine for EBV-positive nasopharyngeal carcinoma. J. Virol..

[97] Lutzky V.P., Corban M., Heslop L., Morrison L.E., Crooks P., Hall D.F., Coman W.B., Thomson S.A., Moss D.J. (2010). Novel approach to the formulation of an Epstein-Barr virus antigen-based nasopharyngeal carcinoma vaccine. J. Virol..

[98] Hancock G., Hellner K., Dorrell L. (2018). Therapeutic HPV vaccines. Best Pract. Res. Clin. Obstet. Gynaecol..

[99] Schiller J.T., Castellsague X., Garland S.M. (2012). A review of clinical trials of human papillomavirus prophylactic vaccines. Vaccine.

[100] Maciag P.C., Radulovic S., Rothman J. (2009). The first clinical use of a live-attenuated Listeria monocytogenes vaccine: A Phase I safety study of Lm-LLO-E7 in patients with advanced carcinoma of the cervix. Vaccine.

[101] Welters M.J., Kenter G.G., Piersma S.J., Vloon A.P., Lowik M.J., Berends-van der Meer D.M., Drijfhout J.W., Valentijn A.R., Wafelman A.R., Oostendorp J. (2008). Induction of tumor-specific CD4+ and CD8+ T-cell immunity in cervical cancer patients by a human papillomavirus type 16 E6 and E7 long peptides vaccine. Clin. Cancer Res..

[102] Aggarwal C., Cohen R.B., Morrow M.P., Kraynyak K.A., Sylvester A.J., Knoblock D.M., Bauml J.M., Weinstein G.S., Lin A., Boyer J. (2019). Immunotherapy Targeting HPV16/18 Generates Potent Immune Responses in HPV-Associated Head and Neck Cancer. Clin. Cancer Res..

[103] Lin C.L., Lo W.F., Lee T.H., Ren Y., Hwang S.L., Cheng Y.F., Chen C.L., Chang Y.S., Lee S.P., Rickinson A.B. (2002). Immunization with Epstein-Barr Virus (EBV) peptide-pulsed dendritic cells induces functional CD8+ T-cell immunity and may lead to tumor regression in patients with EBV-positive nasopharyngeal carcinoma. Cancer Res..

[104] Chia W.K., Wang W.W., Teo M., Tai W.M., Lim W.T., Tan E.H., Leong S.S., Sun L., Chen J.J., Gottschalk S. (2012). A phase II study evaluating the safety and efficacy of an adenovirus-DeltaLMP1-LMP2 transduced dendritic cell vaccine in patients with advanced metastatic nasopharyngeal carcinoma. Ann. Oncol..

[105] Taylor G.S., Jia H., Harrington K., Lee L.W., Turner J., Ladell K., Price D.A., Tanday M., Matthews J., Roberts C. (2014). A recombinant modified vaccinia ankara vaccine encoding Epstein-Barr Virus (EBV) target antigens: A phase I trial in UK patients with EBV-positive cancer. Clin. Cancer Res..

[106] Hui E.P., Taylor G.S., Jia H., Ma B.B., Chan S.L., Ho R., Wong W.L., Wilson S., Johnson B.F., Edwards C. (2013). Phase I trial of recombinant modified vaccinia ankara encoding Epstein-Barr viral tumor antigens in nasopharyngeal carcinoma patients. Cancer Res..

[107] Yutani S., Ueshima K., Abe K., Ishiguro A., Eguchi J., Matsueda S., Komatsu N., Shichijo S., Yamada A., Itoh K. (2015). Phase II Study of Personalized Peptide Vaccination with Both a Hepatitis C Virus-Derived Peptide and Peptides from Tumor-Associated Antigens for the Treatment of HCV-Positive Advanced Hepatocellular Carcinoma Patients. J. Immunol. Res..

[108] Geall A.J., Ulmer J.B. (2015). Introduction to RNA-based vaccines and therapeutics. Expert Rev. Vaccines.

[109] Walsh E.E., Frenck R.W., Falsey A.R., Kitchin N., Absalon J., Gurtman A., Lockhart S., Neuzil K., Mulligan M.J., Bailey R. (2020). Safety and Immunogenicity of Two RNA-Based Covid-19 Vaccine Candidates. N. Engl. J. Med..

[110] Iqbal N., Iqbal N. (2014). Human Epidermal Growth Factor Receptor 2 (HER2) in Cancers: Overexpression and Therapeutic Implications. Mol. Biol. Int..

[111] Pinzani P., Lind K., Malentacchi F., Nesi G., Salvianti F., Villari D., Kubista M., Pazzagli M., Orlando C. (2008). Prostate-specific antigen mRNA and protein levels in laser microdissected cells of human prostate measured by real-time reverse transcriptase-quantitative polymerase chain reaction and immuno-quantitative polymerase chain reaction. Hum. Pathol..

[112] Liu C.C., Yang H., Zhang R., Zhao J.J., Hao D.J. (2017). Tumour-associated antigens and their anti-cancer applications. Eur. J. Cancer Care.

[113] Bright R.K., Bright J.D., Byrne J.A. (2014). Overexpressed oncogenic tumor-self antigens. Hum. Vaccines Immunother..

[114] Haen S.P., Rammensee H.G. (2013). The repertoire of human tumor-associated epitopes—Identification and selection of antigens and their application in clinical trials. Curr. Opin. Immunol..

[115] Bart J., Groen H.J., van der Graaf W.T., Hollema H., Hendrikse N.H., Vaalburg W., Sleijfer D.T., de Vries E.G. (2002). An oncological view on the blood-testis barrier. Lancet. Oncol..

[116] Gjerstorff M.F., Kock K., Nielsen O., Ditzel H.J. (2007). MAGE-A1, GAGE and NY-ESO-1 cancer/testis antigen expression during human gonadal development. Hum. Reprod..

[117] Fiszer D., Kurpisz M. (1998). Major histocompatibility complex expression on human, male germ cells: A review. Am. J. Reprod. Immunol..

[118] Fijak M., Meinhardt A. (2006). The testis in immune privilege. Immunol. Rev..

[119] Fratta E., Coral S., Covre A., Parisi G., Colizzi F., Danielli R., Nicolay H.J., Sigalotti L., Maio M. (2011). The biology of cancer testis antigens: Putative function, regulation and therapeutic potential. Mol. Oncol..

[120] Yao J., Caballero O.L., Yung W.K., Weinstein J.N., Riggins G.J., Strausberg R.L., Zhao Q. (2014). Tumor subtype-specific cancer-testis antigens as potential biomarkers and immunotherapeutic targets for cancers. Cancer Immunol. Res..

[121] Simpson A.J., Caballero O.L., Jungbluth A., Chen Y.T., Old L.J. (2005). Cancer/testis antigens, gametogenesis and cancer. Nat. Rev. Cancer.

[122] Van der Bruggen P., Traversari C., Chomez P., Lurquin C., De Plaen E., Van den Eynde B., Knuth A., Boon T. (1991). A gene encoding an antigen recognized by cytolytic T lymphocytes on a human melanoma. Science.

[123] Almeida L.G., Sakabe N.J., deoliveira A.R., Silva M.C., Mundstein A.S., Cohen T., Chen Y.T., Chua R., Gurung S., Gnjatic S. (2009). CTdatabase: A knowledge-base of high-throughput and curated data on cancer-testis antigens. Nucleic Acids Res..

[124] Chomez P., De Backer O., Bertrand M., De Plaen E., Boon T., Lucas S. (2001). An overview of the MAGE gene family with the identification of all human members of the family. Cancer Res..

[125] Sang M., Lian Y., Zhou X., Shan B. (2011). MAGE-A family: Attractive targets for cancer immunotherapy. Vaccine.

[126] Esfandiary A., Ghafouri-Fard S. (2015). MAGE-A3: An immunogenic target used in clinical practice. Immunotherapy.

[127] Gaugler B., Van den Eynde B., van der Bruggen P., Romero P., Gaforio J.J., De Plaen E., Lethé B., Brasseur F., Boon T. (1994). Human gene MAGE-3 codes for an antigen recognized on a melanoma by autologous cytolytic T lymphocytes. J. Exp. Med..

[128] Van der Bruggen P., Bastin J., Gajewski T., Coulie P.G., Boël P., De Smet C., Traversari C., Townsend A., Boon T. (1994). A peptide encoded by human gene MAGE-3 and presented by HLA-A2 induces cytolytic T lymphocytes that recognize tumor cells expressing MAGE-3. Eur. J. Immunol..

[129] Oiso M., Eura M., Katsura F., Takiguchi M., Sobao Y., Masuyama K., Nakashima M., Itoh K., Ishikawa T. (1999). A newly identified MAGE-3-derived epitope recognized by HLA-A24-restricted cytotoxic T lymphocytes. Int. J. Cancer.

[130] Tanzarella S., Russo V., Lionello I., Dalerba P., Rigatti D., Bordignon C., Traversari C. (1999). Identification of a promiscuous T-cell epitope encoded by multiple members of the MAGE family. Cancer Res..

[131] Schultz E.S., Lethé B., Cambiaso C.L., Van Snick J., Chaux P., Corthals J., Heirman C., Thielemans K., Boon T., van der Bruggen P. (2000). A MAGE-A3 peptide presented by HLA-DP4 is recognized on tumor cells by CD4^+^ cytolytic T lymphocytes. Cancer Res..

[132] Kobayashi H., Song Y., Hoon D.S., Appella E., Celis E. (2001). Tumor-reactive T helper lymphocytes recognize a promiscuous MAGE-A3 epitope presented by various major histocompatibility complex class II alleles. Cancer Res..

[133] Duperret E.K., Liu S., Paik M., Trautz A., Stoltz R., Liu X., Ze K., Perales-Puchalt A., Reed C., Yan J. (2018). A Designer Cross-reactive DNA Immunotherapeutic Vaccine that Targets Multiple MAGE-A Family Members Simultaneously for Cancer Therapy. Clin. Cancer Res..

[134] Bonehill A., Heirman C., Tuyaerts S., Michiels A., Breckpot K., Brasseur F., Zhang Y., Van Der Bruggen P., Thielemans K. (2004). Messenger RNA-electroporated dendritic cells presenting MAGE-A3 simultaneously in HLA class I and class II molecules. J. Immunol..

[135] Liu X., Song N., Liu Y., Liu Y., Li J., Ding J., Tong Z. (2015). Efficient induction of anti-tumor immune response in esophageal squamous cell carcinoma via dendritic cells expressing MAGE-A3 and CALR antigens. Cell. Immunol..

[136] Sartorius R., Pisu P., D’Apice L., Pizzella L., Romano C., Cortese G., Giorgini A., Santoni A., Velotti F., De Berardinis P. (2008). The use of filamentous bacteriophage fd to deliver MAGE-A10 or MAGE-A3 HLA-A2-restricted peptides and to induce strong antitumor CTL responses. J. Immunol..

[137] Zhou D., Zheng H., Liu Q., Lu X., Deng X., Jiang L., Hou B., Fu Y., Zhu F., Ding Y. (2019). Attenuated plasmodium sporozoite expressing MAGE-A3 induces antigen-specific CD8+ T cell response against lung cancer in mice. Cancer Biol. Med..

[138] Batchu R.B., Gruzdyn O., Potti R.B., Weaver D.W., Gruber S.A. (2014). MAGE-A3 with cell-penetrating domain as an efficient therapeutic cancer vaccine. JAMA Surg..

[139] Thomas R., Al-Khadairi G., Roelands J., Hendrickx W., Dermime S., Bedognetti D., Decock J. (2018). NY-ESO-1 Based Immunotherapy of Cancer: Current Perspectives. Front. Immunol..

[140] Hemminger J.A., Ewart Toland A., Scharschmidt T.J., Mayerson J.L., Kraybill W.G., Guttridge D.C., Iwenofu O.H. (2013). The cancer-testis antigen NY-ESO-1 is highly expressed in myxoid and round cell subset of liposarcomas. Mod. Pathol..

[141] Kim S.H., Lee S., Lee C.H., Lee M.K., Kim Y.D., Shin D.H., Choi K.U., Kim J.Y., Park D.Y., Sol M.Y. (2009). Expression of cancer-testis antigens MAGE-A3/6 and NY-ESO-1 in non-small-cell lung carcinomas and their relationship with immune cell infiltration. Lung.

[142] Aung P.P., Liu Y.C., Ballester L.Y., Robbins P.F., Rosenberg S.A., Lee C.C. (2014). Expression of New York esophageal squamous cell carcinoma-1 in primary and metastatic melanoma. Hum. Pathol..

[143] Fosså A., Berner A., Fosså S.D., Hernes E., Gaudernack G., Smeland E.B. (2004). NY-ESO-1 protein expression and humoral immune responses in prostate cancer. Prostate.

[144] Chen Y.T., Scanlan M.J., Sahin U., Türeci O., Gure A.O., Tsang S., Williamson B., Stockert E., Pfreundschuh M., Old L.J. (1997). A testicular antigen aberrantly expressed in human cancers detected by autologous antibody screening. Proc. Natl. Acad. Sci. USA.

[145] Jäger E., Chen Y.T., Drijfhout J.W., Karbach J., Ringhoffer M., Jäger D., Arand M., Wada H., Noguchi Y., Stockert E. (1998). Simultaneous humoral and cellular immune response against cancer-testis antigen NY-ESO-1: Definition of human histocompatibility leukocyte antigen (HLA)-A2-binding peptide epitopes. J. Exp. Med..

[146] Chen J.L., Dunbar P.R., Gileadi U., Jäger E., Gnjatic S., Nagata Y., Stockert E., Panicali D.L., Chen Y.T., Knuth A. (2000). Identification of NY-ESO-1 peptide analogues capable of improved stimulation of tumor-reactive CTL. J. Immunol..

[147] Zarour H.M., Storkus W.J., Brusic V., Williams E., Kirkwood J.M. (2000). NY-ESO-1 encodes DRB1*0401-restricted epitopes recognized by melanoma-reactive CD4+ T cells. Cancer Res..

[148] Chen Y., Huang A., Gao M., Yan Y., Zhang W. (2013). Potential therapeutic value of dendritic cells loaded with NY-ESO-1 protein for the immunotherapy of advanced hepatocellular carcinoma. Int. J. Mol. Med..

[149] Campos-Perez J., Rice J., Escors D., Collins M., Paterson A., Savelyeva N., Stevenson F.K. (2013). DNA fusion vaccine designs to induce tumor-lytic CD8+ T-cell attack via the immunodominant cysteine-containing epitope of NY-ESO 1. Int. J. Cancer.

[150] Delaunay T., Violland M., Boisgerault N., Dutoit S., Vignard V., Münz C., Gannage M., Dréno B., Vaivode K., Pjanova D. (2018). Oncolytic viruses sensitize human tumor cells for NY-ESO-1 tumor antigen recognition by CD4+ effector T cells. Oncoimmunology.

[151] Li M., Shi H., Mu Y., Luo Z., Zhang H., Wan Y., Zhang D., Lu L., Men K., Tian Y. (2014). Effective inhibition of melanoma tumorigenesis and growth via a new complex vaccine based on NY-ESO-1-alum-polysaccharide-HH2. Mol. Cancer.

[152] Tian Y., Li M., Yu C., Zhang R., Zhang X., Huang R., Lu L., Yuan F., Fan Y., Zhou B. (2017). The novel complex combination of alum, CpG ODN and HH2 as adjuvant in cancer vaccine effectively suppresses tumor growth in vivo. Oncotarget.

[153] Ikeda H., Lethé B., Lehmann F., van Baren N., Baurain J.F., de Smet C., Chambost H., Vitale M., Moretta A., Boon T. (1997). Characterization of an antigen that is recognized on a melanoma showing partial HLA loss by CTL expressing an NK inhibitory receptor. Immunity.

[154] Al-Khadairi G., Decock J. (2019). Cancer Testis Antigens and Immunotherapy: Where Do We Stand in the Targeting of PRAME?. Cancers.

[155] Field M.G., Decatur C.L., Kurtenbach S., Gezgin G., van der Velden P.A., Jager M.J., Kozak K.N., Harbour J.W. (2016). PRAME as an Independent Biomarker for Metastasis in Uveal Melanoma. Clin. Cancer Res..

[156] Tan P., Zou C., Yong B., Han J., Zhang L., Su Q., Yin J., Wang J., Huang G., Peng T. (2012). Expression and prognostic relevance of PRAME in primary osteosarcoma. Biochem. Biophys. Res. Commun..

[157] Iura K., Kohashi K., Hotokebuchi Y., Ishii T., Maekawa A., Yamada Y., Yamamoto H., Iwamoto Y., Oda Y. (2015). Cancer-testis antigens PRAME and NY-ESO-1 correlate with tumour grade and poor prognosis in myxoid liposarcoma. J. Pathol. Clin. Res..

[158] Rezvani K., Yong A.S., Tawab A., Jafarpour B., Eniafe R., Mielke S., Savani B.N., Keyvanfar K., Li Y., Kurlander R. (2009). Ex vivo characterization of polyclonal memory CD8+ T-cell responses to PRAME-specific peptides in patients with acute lymphoblastic leukemia and acute and chronic myeloid leukemia. Blood.

[159] Gérard C., Baudson N., Ory T., Segal L., Louahed J. (2015). A Comprehensive Preclinical Model Evaluating the Recombinant PRAME Antigen Combined With the AS15 Immunostimulant to Fight Against PRAME-expressing Tumors. J. Immunother..

[160] Epping M.T., Wang L., Edel M.J., Carlée L., Hernandez M., Bernards R. (2005). The human tumor antigen PRAME is a dominant repressor of retinoic acid receptor signaling. Cell.

[161] Vansteenkiste J.F., Cho B.C., Vanakesa T., De Pas T., Zielinski M., Kim M.S., Jassem J., Yoshimura M., Dahabreh J., Nakayama H. (2016). Efficacy of the MAGE-A3 cancer immunotherapeutic as adjuvant therapy in patients with resected MAGE-A3-positive non-small-cell lung cancer (MAGRIT): A randomised, double-blind, placebo-controlled, phase 3 trial. Lancet Oncol..

[162] Dreno B., Thompson J.F., Smithers B.M., Santinami M., Jouary T., Gutzmer R., Levchenko E., Rutkowski P., Grob J.-J., Korovin S. (2018). MAGE-A3 immunotherapeutic as adjuvant therapy for patients with resected, MAGE-A3-positive, stage III melanoma (DERMA): A double-blind, randomised, placebo-controlled, phase 3 trial. Lancet Oncol..

[163] Krishnadas D.K., Shusterman S., Bai F., Diller L., Sullivan J.E., Cheerva A.C., George R.E., Lucas K.G. (2015). A phase I trial combining decitabine/dendritic cell vaccine targeting MAGE-A1, MAGE-A3 and NY-ESO-1 for children with relapsed or therapy-refractory neuroblastoma and sarcoma. Cancer Immunol. Immunother. CII.

[164] Gasser O., Sharples K.J., Barrow C., Williams G.M., Bauer E., Wood C.E., Mester B., Dzhelali M., Caygill G., Jones J. (2018). A phase I vaccination study with dendritic cells loaded with NY-ESO-1 and alpha-galactosylceramide: Induction of polyfunctional T cells in high-risk melanoma patients. Cancer Immunol. Immunother. CII.

[165] Westdorp H., Creemers J.H.A., van Oort I.M., Schreibelt G., Gorris M.A.J., Mehra N., Simons M., de Goede A.L., van Rossum M.M., Croockewit A.J. (2019). Blood-derived dendritic cell vaccinations induce immune responses that correlate with clinical outcome in patients with chemo-naive castration-resistant prostate cancer. J. Immunother. Cancer.

[166] Pujol J.L., De Pas T., Rittmeyer A., Vallieres E., Kubisa B., Levchenko E., Wiesemann S., Masters G.A., Shen R., Tjulandin S.A. (2016). Safety and Immunogenicity of the PRAME Cancer Immunotherapeutic in Patients with Resected Non-Small Cell Lung Cancer: A Phase I Dose Escalation Study. J. Thorac. Oncol..

[167] Weber J.S., Vogelzang N.J., Ernstoff M.S., Goodman O.B., Cranmer L.D., Marshall J.L., Miles S., Rosario D., Diamond D.C., Qiu Z. (2011). A phase 1 study of a vaccine targeting preferentially expressed antigen in melanoma and prostate-specific membrane antigen in patients with advanced solid tumors. J. Immunother..

[168] Dalgleish A.G. (2015). Rationale for combining immunotherapy with chemotherapy. Immunotherapy.

[169] Gordeeva O. (2018). Cancer-testis antigens: Unique cancer stem cell biomarkers and targets for cancer therapy. Semin. Cancer Biol..

[170] Fukuda K., Funakoshi T., Sakurai T., Nakamura Y., Mori M., Tanese K., Tanikawa A., Taguchi J., Fujita T., Okamoto M. (2017). Peptide-pulsed dendritic cell vaccine in combination with carboplatin and paclitaxel chemotherapy for stage IV melanoma. Melanoma Res..

[171] Chen X., Wang L., Li P., Song M., Qin G., Gao Q., Zhang Z., Yue D., Wang D., Nan S. (2018). Dual TGF-β and PD-1 blockade synergistically enhances MAGE-A3-specific CD8(+) T cell response in esophageal squamous cell carcinoma. Int. J. Cancer.

[172] Fourcade J., Sun Z., Benallaoua M., Guillaume P., Luescher I.F., Sander C., Kirkwood J.M., Kuchroo V., Zarour H.M. (2010). Upregulation of Tim-3 and PD-1 expression is associated with tumor antigen-specific CD8+ T cell dysfunction in melanoma patients. J. Exp. Med..

[173] Matsuzaki J., Gnjatic S., Mhawech-Fauceglia P., Beck A., Miller A., Tsuji T., Eppolito C., Qian F., Lele S., Shrikant P. (2010). Tumor-infiltrating NY-ESO-1-specific CD8+ T cells are negatively regulated by LAG-3 and PD-1 in human ovarian cancer. Proc. Natl. Acad. Sci. USA.

[174] Yuan J., Gnjatic S., Li H., Powel S., Gallardo H.F., Ritter E., Ku G.Y., Jungbluth A.A., Segal N.H., Rasalan T.S. (2008). CTLA-4 blockade enhances polyfunctional NY-ESO-1 specific T cell responses in metastatic melanoma patients with clinical benefit. Proc. Natl. Acad. Sci. USA.

[175] Dos Santos L.I., Galvao-Filho B., de Faria P.C., Junqueira C., Dutra M.S., Teixeira S.M., Rodrigues M.M., Ritter G., Bannard O., Fearon D.T. (2015). Blockade of CTLA-4 promotes the development of effector CD8+ T lymphocytes and the therapeutic effect of vaccination with an attenuated protozoan expressing NY-ESO-1. Cancer Immunol. Immunother. CII.

[176] Gibney G.T., Kudchadkar R.R., DeConti R.C., Thebeau M.S., Czupryn M.P., Tetteh L., Eysmans C., Richards A., Schell M.J., Fisher K.J. (2015). Safety, correlative markers, and clinical results of adjuvant nivolumab in combination with vaccine in resected high-risk metastatic melanoma. Clin. Cancer Res..

[177] Sigalotti L., Coral S., Fratta E., Lamaj E., Danielli R., Di Giacomo A.M., Altomonte M., Maio M. (2005). Epigenetic modulation of solid tumors as a novel approach for cancer immunotherapy. Semin. Oncol..

[178] Lyko F., Brown R. (2005). DNA methyltransferase inhibitors and the development of epigenetic cancer therapies. J. Natl. Cancer Inst..

[179] Bao L., Dunham K., Lucas K. (2011). MAGE-A1, MAGE-A3, and NY-ESO-1 can be upregulated on neuroblastoma cells to facilitate cytotoxic T lymphocyte-mediated tumor cell killing. Cancer Immunol. Immunother. CII.

[180] Odunsi K., Matsuzaki J., James S.R., Mhawech-Fauceglia P., Tsuji T., Miller A., Zhang W., Akers S.N., Griffiths E.A., Miliotto A. (2014). Epigenetic potentiation of NY-ESO-1 vaccine therapy in human ovarian cancer. Cancer Immunol. Res..

[181] Golovastova M.O., Bazhin A.V., Philippov P.P. (2014). Cancer-retina antigens—A new group of tumor antigens. Biochem. Biochim..

[182] Bazhin A.V., Schadendorf D., Willner N., De Smet C., Heinzelmann A., Tikhomirova N.K., Umansky V., Philippov P.P., Eichmuller S.B. (2007). Photoreceptor proteins as cancer-retina antigens. Int. J. Cancer.

[183] Baldin A.V., Grishina A.N., Korolev D.O., Kuznetsova E.B., Golovastova M.O., Kalpinskiy A.S., Alekseev B.Y., Kaprin A.D., Zinchenko D.V., Savvateeva L.V. (2019). Autoantibody against arrestin-1 as a potential biomarker of renal cell carcinoma. Biochimie.

[184] Milam A.H., Saari J.C., Jacobson S.G., Lubinski W.P., Feun L.G., Alexander K.R. (1993). Autoantibodies against retinal bipolar cells in cutaneous melanoma-associated retinopathy. Investig. Ophthalmol. Vis. Sci..

[185] Baldin A.V., Savvateeva L.V., Bazhin A.V., Zamyatnin A.A. (2020). Dendritic Cells in Anticancer Vaccination: Rationale for Ex Vivo Loading or In Vivo Targeting. Cancers.

[186] Baldin A.V., Zamyatnin A.A., Bazhin A.V., Xu W.H., Savvateeva L.V. (2019). Advances in the Development of Anticancer HSP-based Vaccines. Curr. Med. Chem..

