# Supplementary material for: Cancer Vaccines: Antigen Selection Strategy
Source: Vaccines (Basel). 2021 Jan 25;9(2):85. doi: 10.3390/vaccines9020085 (PMC7911511; doi:10.3390/vaccines9020085)
Supplement: Supplementary file 1 [file vaccines-09-00085-s001.pdf]

Table S1. Selective clinical trials of viral antigens-based cancer vaccines.

Type of Intervention	Target Markers	Disease	Phase	Tiral ID	Patients Number	Clinical Benefit (Patients Number)	Serious Adverse Events (Patients Numbers)	Other Intervention	OS (Months)	PFS (Months)	Specific T Cells re-Sponse (Positive/Test Number)	Ref.	Publish Year
Peptide	HPV 16 E6/E7	CC	II	NA	6	NA	No Grade ≥3 events	None	13	NA	6/6	[1]	2008
Listeria Monocytogenes	HPV 16 E7	CC	I	NA	15	SD (7) PR (1)	Grade 3 (6)	None	11.6	NA	1/3	[2]	2009
DNA Vaccine	HPV 16/18 E6/E7	HNSCC	I/IIa	NCT02163057	22	NA	No Grade ≥3 events	None	15.9	12-month DFS rate (89.4%)	8/21	[3]	2018
Dendritic Cell	LMP2	NPC	I	NA	16	PR (2)	No Grade ≥3 events	None	NA	NA	9/16	[4]	2002
Dendritic Cell	LMP1, LMP2	NPC	II	NA	16	SD (2) PR (1)	Grade 3 (1)	None	6	1.92	0/8	[5]	2012
Virus	EBNA1, LMP2	NPC	I	NCT01256853	18	NA	Grade 3 (1)	None	NA	NA	15/18	[6]	2013
Virus	EBNA1, LMP2	NPC	Ia	NCT01147991	16	NA	No Grade ≥3 events	None	NA	NA	8/14	[7]	2014
Peptide	HCV Core	HCC	II	UMIN000003520 UMIN000005634	42	NA	Total events: Grade 3 (14), Grade 5 (1)	Sorafenib	6.1	NA	19/36	[8]	2015

OS: overall survival; PFS: progression-free survival; HPV: Human papillomavirus; LMP: latent membrane proteins; EBNA1: Epstein-Barr virus nuclear antigen 1; HCV: hepatitis C virus; CC: Cervical cancer; HNSCC: Head and neck squamous cell cancer; NPC: Nasopharyngeal carcinoma; HCC: hepatocellular carcinoma; CR: complete response; PR: partial response; SD: stable disease; NA: not applicable.

Table S2. Selective clinical trials of CGAs-based cancer vaccines.

Type of intervention	Target	Disease	Phase	Patients number	Clinical benefit	Serious adverse events	Other intervention	OS (months)	PFS (Months)	Specific T cells response	Trial ID	Ref.

					(patients number)	(patients numbers)				(positive/test number)		
Recombinant Protein	MAGE-A3	Melanoma	II	18	SD (6) PR (1) CR (3)	Grade 3 (1)	High-dose interleukin-2 (HDIL-2)	NA	3.6	NA	NCT01266603	[9]
DC	MAGE-A3, MAGE-A2, WT1, gp100, tyrosinase	Melanoma	I/II	9	SD (4) PR (1)	Grade 3 (9) Grade 4 (1)	Carboplatin Paclitaxel	12	2.3	5/9	UMIN000006629	[10]
Recombinant Protein	MAGE-A3	BC	I	24	NA	NA	Bacillus-Calmette-Guerin	NA	NA	12/23	NCT01498172	[11]
Recombinant Protein	MAGE-A3	NSCLC	I	67	NA	Grade 3/4 (24) Grade 5 (1)	Adjuvant Chemotherapy	NA	NA	CD4+:15/40 CD8+:4/40	NCT00455572	[12]
DC	MAGE-A1, MAGE-A3, NY-ESO-1	Neuroblastoma and sarcoma	I	10	SD (1) CR (1)	Grade 3 (1) Grade 4 (4)	Decitabine	NA	NA	6/9	NCT01241162	[13]
Recombinant Protein	MAGE-A3	NSCLC	II	122	NA	Grade 3 (13) Grade 4 (2)	None	57	45	NA	NCT00290355	[14]
Recombinant Protein	MAGE-A3	Melanoma	II	72	SD (10) PR (2) CR (3)	Grade 3 (3)	None	AS15 arm: 33 AS02B arm: 19	AS15 arm: 11.6 AS02B arm: 7.1	AS15 arm: 18/26 AS02B arm: 6/29	NCT00086866	[15]
Recombinant Protein	NY-ESO-1	STs	I	26	SD (8)	Grade 3 (6)	None	NA	NA	2/8	UMIN000005246 UMIN000008006	[16]

DC	NY-ESO-1, MAGE-C2, MUC1	PC	IIa	21	SD (12) PR (1)	No Grade ≥3 events	None	NA	9.5	12/21	NCT02692976	[17]
Recombinant Protein	NY-ESO-1	Melanoma	I/II	35	NA	Grade 3 (1)	Montanide	NA	NA	CD4+:18/21 CD8+:4/21	NYU#09–0007 MSSM#13–1391	[18]
Lentivirus Vector	NY-ESO-1	STs	I	39	SD (20) PR (2)	No Grade ≥3 events	None	31.1	4.6	17/33	NCT02122861	[19]
Recombinant Protein	NY-ESO-1	STs	I	13	SD (3)	Grade 3 (1)	None	NA	NA	CD4+:6/11 CD8+:4/11	NCT02015416	[20]
mRNA	NY-ESO-1, MAGE-C1, MAGE-C2, survivin, 5T4, MUC-1	NSCLC	Ib	26	SD (12) PR (1)	Grade ≥3 (4)	Pemetrexed EGFR-TKI	13.95	2.87	10/25	NCT01915524	[21]
Peptide	NY-ESO-1 gp100 MART-1	Melanoma	I	33	NA	Grade 3–5 (9)	Nivolumab	NA	47.1	NA	NA	[22]
Recombinant Protein	NY-ESO-1	OC	I	12	SD (5) PR (1)	Grade 3 (2)	decitabine	NA	NA	7/11	NCT00887796	[23]
Recombinant Protein	PRAME	NSCLC	I	60	NA	No Grade ≥3 events	None	NA	NA	CD4+:26/35 CD8+:2/32	NCT01159964	[24]
Peptide	PRAME, PSMA	STs	I	26	SD (10)	No Grade ≥3 events	None	NA	NA	15/24	NA	[25]

OS: overall survival; PFS: progression-free survival; MAGE-A3: melanoma-associated antigen A3; WT1: wilms' tumor protein; gp100: glycoprotein 100; MUC1: mucin 1; NY-ESO-1: New York esophageal squamous cell carcinoma-1; PRAME: preferentially expressed antigen in melanoma; PSMA: prostate-specific membrane antigen; BC: bladder cancer; NSCLC: non-small cell lung cancer; STs: solid tumors; PC: prostate cancer; OC: ovarian cancer; CR: complete response; PR: partial response; SD: stable disease; EGFR-TKI: epidermal growth factor receptor -tyrosine kinase inhibitor; NA: not applicable.

Reference

1. Welters, M.J.; Kenter, G.G.; Piersma, S.J.; Vloon, A.P.; Lowik, M.J.; Berends-van der Meer, D.M.; Drijfhout, J.W.; Valentijn, A.R.; Wafelman, A.R.; Oostendorp, J.; et al. Induction of tumor-specific CD4+ and CD8+ T-cell immunity in cervical cancer patients by a human papillomavirus type 16 E6 and E7 long peptides vaccine. *Clin. Cancer Res.* **2008**, *14*, 178–187, doi:10.1158/1078-0432.CCR-07-1880.
2. Maciag, P.C.; Radulovic, S.; Rothman, J. The first clinical use of a live-attenuated Listeria monocytogenes vaccine: A Phase I safety study of Lm-LLO-E7 in patients with advanced carcinoma of the cervix. *Vaccine* **2009**, *27*, 3975–3983, doi:10.1016/j.vaccine.2009.04.041.
3. Aggarwal, C.; Cohen, R.B.; Morrow, M.P.; Kraynyak, K.A.; Sylvester, A.J.; Knoblock, D.M.; Bauml, J.M.; Weinstein, G.S.; Lin, A.; Boyer, J.; et al. Immunotherapy Targeting HPV16/18 Generates Potent Immune Responses in HPV-Associated Head and Neck Cancer. *Clin. Cancer Res.* **2019**, *25*, 110–124, doi:10.1158/1078-0432.CCR-18-1763.
4. Lin, C.L.; Lo, W.F.; Lee, T.H.; Ren, Y.; Hwang, S.L.; Cheng, Y.F.; Chen, C.L.; Chang, Y.S.; Lee, S.P.; Rickinson, A.B.; et al. Immunization with Epstein-Barr Virus (EBV) peptide-pulsed dendritic cells induces functional CD8+ T-cell immunity and may lead to tumor regression in patients with EBV-positive nasopharyngeal carcinoma. *Cancer Res.* **2002**, *62*, 6952–6958.
5. Chia, W.K.; Wang, W.W.; Teo, M.; Tai, W.M.; Lim, W.T.; Tan, E.H.; Leong, S.S.; Sun, L.; Chen, J.J.; Gottschalk, S.; et al. A phase II study evaluating the safety and efficacy of an adenovirus-DeltaLMP1-LMP2 transduced dendritic cell vaccine in patients with advanced metastatic nasopharyngeal carcinoma. *Ann. Oncol.* **2012**, *23*, 997–1005, doi:10.1093/annonc/mdr341.
6. Hui, E.P.; Taylor, G.S.; Jia, H.; Ma, B.B.; Chan, S.L.; Ho, R.; Wong, W.L.; Wilson, S.; Johnson, B.F.; Edwards, C.; et al. Phase I trial of recombinant modified vaccinia ankara encoding Epstein-Barr viral tumor antigens in nasopharyngeal carcinoma patients. *Cancer Res.* **2013**, *73*, 1676–1688, doi:10.1158/0008-5472.CAN-12-2448.
7. Taylor, G.S.; Jia, H.; Harrington, K.; Lee, L.W.; Turner, J.; Ladell, K.; Price, D.A.; Tanday, M.; Matthews, J.; Roberts, C.; et al. A recombinant modified vaccinia ankara vaccine encoding Epstein-Barr Virus (EBV) target antigens: A phase I trial in UK patients with EBV-positive cancer. *Clin. Cancer Res.* **2014**, *20*, 5009–5022, doi:10.1158/1078-0432.CCR-14-1122-T.
8. Yutani, S.; Ueshima, K.; Abe, K.; Ishiguro, A.; Eguchi, J.; Matsueda, S.; Komatsu, N.; Shichijo, S.; Yamada, A.; Itoh, K.; et al. Phase II Study of Personalized Peptide Vaccination with Both a Hepatitis C Virus-Derived Peptide and Peptides from Tumor-Associated Antigens for the Treatment of HCV-Positive Advanced Hepatocellular Carcinoma Patients. *J. Immunol. Res.* **2015**, *2015*, 473909, doi:10.1155/2015/473909.
9. McQuade, J.L.; Homsi, J.; Torres-Cabala, C.A.; Bassett, R.; Popuri, R.M.; James, M.L.; Vence, L.M.; Hwu, W.J. A phase II trial of recombinant MAGE-A3 protein with immunostimulant AS15 in combination with high-dose Interleukin-2 (HDIL2) induction therapy in metastatic melanoma. *BMC cancer* **2018**, *18*, 1274, doi:10.1186/s12885-018-5193-9.
10. Fukuda, K.; Funakoshi, T.; Sakurai, T.; Nakamura, Y.; Mori, M.; Tanese, K.; Tanikawa, A.; Taguchi, J.; Fujita, T.; Okamoto, M.; et al. Peptide-pulsed dendritic cell vaccine in combination with carboplatin and paclitaxel chemotherapy for stage IV melanoma. *Melanoma Res.* **2017**, *27*, 326–334, doi:10.1097/CMR.0000000000000342.
11. Derre, L.; Cesson, V.; Lucca, I.; Cerantola, Y.; Valerio, M.; Fritschi, U.; Vlamopoulos, Y.; Burruni, R.; Legris, A.S.; Dartiguenave, F.; et al. Intravesical Bacillus Calmette Guerin Combined with a Cancer Vaccine Increases Local T-Cell Responses in Non-muscle-Invasive Bladder Cancer Patients. *Clin. Cancer Res.* **2017**, *23*, 717–725, doi:10.1158/1078-0432.CCR-16-1189.
12. Pujol, J.L.; Vansteenkiste, J.F.; De Pas, T.M.; Atanackovic, D.; Reck, M.; Thomeer, M.; Douillard, J.Y.; Fasola, G.; Potter, V.; Taylor, P.; et al. Safety and Immunogenicity of MAGE-A3 Cancer Immunotherapeutic with or without Adjuvant Chemotherapy in Patients with Resected Stage IB to III MAGE-A3-Positive Non-Small-Cell Lung Cancer. *J. Thorac Oncol* **2015**, *10*, 1458–1467, doi:10.1097/JTO.0000000000000653.
13. Krishnadas, D.K.; Shusterman, S.; Bai, F.; Diller, L.; Sullivan, J.E.; Cheerva, A.C.; George, R.E.; Lucas, K.G. A phase I trial combining decitabine/dendritic cell vaccine targeting MAGE-A1, MAGE-A3 and NY-ESO-1 for children with relapsed or therapy-refractory neuroblastoma and sarcoma. *Cancer Immunol. Immunother.* **2015**, *64*, 1251–1260, doi:10.1007/s00262-015-1731-3.
14. Vansteenkiste, J.; Zielinski, M.; Linder, A.; Dahabreh, J.; Gonzalez, E.E.; Malinowski, W.; Lopez-Brea, M.; Vanakesa, T.; Jassem, J.; Kalofonos, H.; et al. Adjuvant MAGE-A3 immunotherapy in resected non-small-cell lung cancer: Phase II randomized study results. *J. Clin. Oncol.* **2013**, *31*, 2396–2403, doi:10.1200/JCO.2012.43.7103.
15. Kruit, W.H.; Suciu, S.; Dreno, B.; Mortier, L.; Robert, C.; Chiarion-Sileni, V.; Maio, M.; Testori, A.; Dorval, T.; Grob, J.J.; et al. Selection of immunostimulant AS15 for active immunization with MAGE-A3 protein: Results of a randomized phase II study of the European Organisation for Research and Treatment of Cancer Melanoma Group in Metastatic Melanoma. *J. Clin. Oncol.* **2013**, *31*, 2413–2420, doi:10.1200/JCO.2012.43.7111.
16. Ishihara, M.; Tono, Y.; Miyahara, Y.; Muraoka, D.; Harada, N.; Kageyama, S.; Sasaki, T.; Hori, Y.; Soga, N.; Uchida, K.; et al. First-in-human phase I clinical trial of the NY-ESO-1 protein cancer vaccine with NOD2 and TLR9 stimulants in patients with NY-ESO-1-expressing refractory solid tumors. *Cancer Immunol. Immunother.* **2020**, *69*, 663–675, doi:10.1007/s00262-020-02483-1.

17. Westdorp, H.; Creemers, J.H.A.; van Oort, I.M.; Schreibelt, G.; Gorris, M.A.J.; Mehra, N.; Simons, M.; de Goede, A.L.; van Rossum, M.M.; Croockewit, A.J.; et al. Blood-derived dendritic cell vaccinations induce immune responses that correlate with clinical outcome in patients with chemo-naive castration-resistant prostate cancer. *J. Immunother. Cancer* **2019**, *7*, 302, doi:10.1186/s40425-019-0787-6.
18. Pavlick, A.; Blazquez, A.B.; Meseck, M.; Lattanzi, M.; Ott, P.A.; Marron, T.U.; Holman, R.M.; Mandeli, J.; Salazar, A.M.; McClain, C.B.; et al. Combined Vaccination with NY-ESO-1 Protein, Poly-ICLC, and Montanide Improves Humoral and Cellular Immune Responses in Patients with High-Risk Melanoma. *Cancer Immunol. Res.* **2020**, *8*, 70–80, doi:10.1158/2326-6066.CIR-19-0545.
19. Somaiah, N.; Block, M.S.; Kim, J.W.; Shapiro, G.I.; Do, K.T.; Hwu, P.; Eder, J.P.; Jones, R.L.; Lu, H.; Ter Meulen, J.H.; et al. First-in-Class, First-in-Human Study Evaluating LV305, a Dendritic-Cell Tropic Lentiviral Vector, in Sarcoma and Other Solid Tumors Expressing NY-ESO-1. *Clin. Cancer Res.* **2019**, *25*, 5808–5817, doi:10.1158/1078-0432.CCR-19-1025.
20. Mahipal, A.; Ejadi, S.; Gnjatic, S.; Kim-Schulze, S.; Lu, H.; Ter Meulen, J.H.; Kenney, R.; Odunsi, K. First-in-human phase 1 dose-escalating trial of G305 in patients with advanced solid tumors expressing NY-ESO-1. *Cancer Immunol. Immunother.* **2019**, *68*, 1211–1222, doi:10.1007/s00262-019-02331-x.
21. Papachristofilou, A.; Hipp, M.M.; Klinkhardt, U.; Fruh, M.; Sebastian, M.; Weiss, C.; Pless, M.; Cathomas, R.; Hilbe, W.; Pall, G.; et al. Phase Ib evaluation of a self-adjuvanted protamine formulated mRNA-based active cancer immunotherapy, BI1361849 (CV9202), combined with local radiation treatment in patients with stage IV non-small cell lung cancer. *J. Immunother. Cancer* **2019**, *7*, 38, doi:10.1186/s40425-019-0520-5.
22. Gibney, G.T.; Kudchadkar, R.R.; DeConti, R.C.; Thebeau, M.S.; Czupryn, M.P.; Tetteh, L.; Eysmans, C.; Richards, A.; Schell, M.J.; Fisher, K.J.; et al. Safety, correlative markers, and clinical results of adjuvant nivolumab in combination with vaccine in resected high-risk metastatic melanoma. *Clin. Cancer Res.* **2015**, *21*, 712–720, doi:10.1158/1078-0432.Ccr-14-2468.
23. Odunsi, K.; Matsuzaki, J.; James, S.R.; Mhawech-Fauceglia, P.; Tsuji, T.; Miller, A.; Zhang, W.; Akers, S.N.; Griffiths, E.A.; Miliotto, A.; et al. Epigenetic potentiation of NY-ESO-1 vaccine therapy in human ovarian cancer. *Cancer Immunol. Res.* **2014**, *2*, 37–49, doi:10.1158/2326-6066.Cir-13-0126.
24. Pujol, J.L.; De Pas, T.; Rittmeyer, A.; Vallieres, E.; Kubisa, B.; Levchenko, E.; Wiesemann, S.; Masters, G.A.; Shen, R.; Tjulandin, S.A.; et al. Safety and Immunogenicity of the PRAME Cancer Immunotherapeutic in Patients with Resected Non-Small Cell Lung Cancer: A Phase I Dose Escalation Study. *J. Thorac. Oncol* **2016**, *11*, 2208–2217, doi:10.1016/j.jtho.2016.08.120.
25. Weber, J.S.; Vogelzang, N.J.; Ernstoff, M.S.; Goodman, O.B.; Cranmer, L.D.; Marshall, J.L.; Miles, S.; Rosario, D.; Diamond, D.C.; Qiu, Z.; et al. A phase 1 study of a vaccine targeting preferentially expressed antigen in melanoma and prostate-specific membrane antigen in patients with advanced solid tumors. *J Immunol.* **2011**, *34*, 556–567, doi:10.1097/CJI.0b013e3182280db1.
